# Synergistic Effects of Erzhi Pill Combined With Methotrexate on Osteoblasts Mediated *via* the Wnt1/LRP5/*β*-Catenin Signaling Pathway in Collagen-Induced Arthritis Rats

**DOI:** 10.3389/fphar.2020.00228

**Published:** 2020-03-11

**Authors:** Xiaoya Li, Xiangcheng Lu, Danping Fan, Li Li, Cheng Lu, Yong Tan, Ya Xia, Hongyan Zhao, Miaoxuan Fan, Cheng Xiao

**Affiliations:** ^1^Department of Emergency, China-Japan Friendship Hospital, Beijing, China; ^2^Institute of Clinical Medicine, China-Japan Friendship Hospital, Beijing, China; ^3^Graduate School of Peking Union Medical College, Chinese Academy of Medical Sciences/Peking Union Medical College, Beijing, China; ^4^School of Traditional Chinese Medicine, Beijing University of Chinese Medicine, Beijing, China; ^5^Institute of Basic Research in Clinical Medicine, China Academy of Chinese Medical Sciences, Beijing, China; ^6^Beijing Key Laboratory of Research of Chinese Medicine on Prevention and Treatment for Major Diseases, Experimental Research Center, China Academy of Chinese Medical Sciences, Beijing, China; ^7^Beijing Institute for Drug Control, NMPA Key Laboratory for Quality Evaluation of Traditional Chinese Medicine (Traditional Chinese Patent Medicine), Beijing Key Laboratory of Analysis and Evaluation on Chinese Medicine, Beijing, China

**Keywords:** rheumatoid arthritis, Erzhi Pill, Methotrexate, Wnt/*β*-catenin signaling pathway, Osteoblasts, synergistic effects

## Abstract

Rheumatoid arthritis (RA) is a chronic systemic autoimmune disease characterized by chronic synovitis, bone erosion, and bone loss. Erzhi Pill (EZP), a classic Chinese patent medicine, is often used to treat osteoporosis and shows a capacity for bone metabolism regulation. Methotrexate (MTX), an essential drug for RA treatment, has been reported to inhibit generalized bone loss in RA patients. However, the combined therapeutic effects and mechanism of EZP and MTX in RA have not been fully elucidated. The aim of this study was to investigate the synergistic effect of EZP and MTX on RA and to explore the underlying mechanism through network pharmacological prediction and experimental verification. Chemical compounds of EZP, human target proteins of EZP and MTX, and RA-related human genes were identified in the Encyclopedia of Traditional Chinese Medicine database, PubChem database, and NCBI database, respectively. The molecular network of EZP and MTX in RA was generated and analyzed with Ingenuity Pathway Analysis software according to the datasets. Then, MTX monotherapy, EZP monotherapy, and combined MTX and EZP therapy were administered to collagen-induced arthritis rats, followed by assessment of pathological score, bone damage, bone alkaline phosphatases (BALP), and tartrate-resistant acid phosphatase (TRACP), and of gene levels related to the Wnt1/LRP5/*β*-catenin pathway according to network pharmacological analysis. Finally, serum samples from MTX-, EZP- and MTX+EZP-treated rats were used to treat the rat osteoblast (OB)-like UMR-106 cell line to evaluate gene levels related to Wnt1/LRP5/*β*-catenin. Network pharmacological analysis showed that the Wnt/*β*-catenin signaling pathway was the top signaling pathway shared among MTX, EZP, and RA. The results from *in vivo* experiments indicated that EZP combined with MTX reduced arthritis severity, alleviated ankle bone damage, increased BALP and decreased TRACP serum levels, and regulated the mRNA expression of Wnt1, LRP5, *β*-catenin, Runx2, BALP, and BGP in the ankles. *In vitro* experiments showed that EZP combined with MTX could also improve the expression of genes related to the Wnt1/LRP5/*β*-catenin pathway. This study demonstrated that EZP in combination with MTX played a synergistic role in regulating OBs in RA, which was connected to the modulatory effect of EZP and MTX on the Wnt1/LRP5/*β*-catenin signaling pathway.

## Introduction

Rheumatoid arthritis (RA), a clinically common and refractory disease, is pathologically characterized by chronic synovitis of the joints and disrupted bone homeostasis. In the pathological condition of RA, bone homeostasis involving bone formation mediated by osteoblasts (OBs) and bone resorption regulated by osteoclasts (OCs) is disrupted. A previous study showed that the Wnt/*β*-catenin pathway plays an important role in bone development and homeostasis. It can lead to OBs commitment, proliferation, and differentiation, as well as enhancing OBs and osteocyte survival (Glass et al., 2005). Bone destruction, which occurs in RA, is regulated by receptor activator of nuclear factor-κB (RANK) and nuclear factor-kappa beta receptor living factor ligand (RANKL) (Jung et al., 2014). This evidence implies that RA is even more complex than initially predicted.

Erzhi Pill (EZP), a classic Chinese patent medicine consisting of *Ligustrum lucidum Ait*. and *Eclipta prostrata L*. in a one-to-one ratio, is used to clinically improve osteoporosis (OP) in China. One study showed that EZP had potential anti-OP effects by preventing the degradation of the alveolar trabecular microarchitecture and alveolar bone loss in ovariectomized rats through activation of the Wnt3a/low-density lipoprotein receptor-related protein 5 (LRP5)/*β*-catenin signaling pathway (Sun et al., 2014). A study by Liang et al. found that EZP played a therapeutic role in an ovariectomized rat model by improving bone metabolism disorder, bone morphology, bone mineral density (BMD) and bone biomechanics and that the underlying mechanism was related to the Sirt1/Foxo signal (Liang et al., 2018). In addition, an *in vitro* study found that EZP-containing serum could inhibit the proliferation and differentiation of OCs from RAW264.7 cells induced by RANKL (Zhang et al., 2008). However, there are no relevant studies on the application of EZP in the treatment of RA.

Methotrexate (MTX), which is a cornerstone drug for the treatment of RA and has a clinical effective rate of approximately 60%, mainly inhibits inflammation and plays a certain role in bone protection by regulating RANK/RANKL/osteoprotegerin (OPG) (Swierkot et al., 2015; Kanagawa et al., 2016). In addition, several studies have also reported that MTX could improve the expression and activity of OB-related proteins, such as bone alkaline phosphatases (BALP), in primary human OB-like cells (Davies et al., 2002; Davies et al., 2003). To improve its pharmaceutical effect, MTX is often combined with other drugs to treat RA. Takeuchi et al. combined AMG 162 (denosumab) with MTX in a phase II clinical trial, demonstrating an inhibitory effect on the progression of bone erosion at 12 months (Takeuchi et al., 2016). Currently, there is no drug that can be combined with MTX to improve osteogenesis for the treatment of RA. In the clinic, combinations of several drugs that interact with multiple targets in the molecular networks of a disease may achieve better efficacy than monotherapies. Thus, drug combinations can have a synergistic effect without increased toxicity (He et al., 2016).

Considering the limited therapeutic effect of MTX and the multicomponent, multitarget, and multipathway nature of the TCM formula EZP, MTX and EZP were combined, and a network pharmacological approach was applied to explore the pharmacodynamics and corresponding mechanisms of MTX combined with EZP in the treatment of RA. Animal and cell experiments were executed to verify the network pharmacological analysis results. This study may lay the foundation for an optimized combination of drugs treating RA and generate a new approach for treating RA.

## Materials and Methods

### Network Pharmacology-Based Analysis of MTX, EZP, and RA

The chemical compounds of *Ligustrum lucidum Ait*. and *Eclipta prostrata L*., which constitute EZP, were found in the Encyclopedia of Traditional Chinese Medicine (ETCM) database (http://www.ehbio.com/ETCM/). Human protein targets of the above chemical compounds and MTX were retrieved from PubChem Compound (https://pubchem.ncbi.nlm.nih.gov/). Human genes related to RA were obtained from the National Center for Biotechnology Information (NCBI) Gene database (http://www.ncbi.nlm.nih.gov/gene), and “rheumatoid arthritis” was used as a keyword in the Gene database search.

The human target proteins and genes obtained in the first step were then uploaded to the Ingenuity Pathway Analysis (IPA) platform. The molecules imputed to the IPA platform were termed “focus molecules.” IPA generated a set of networks based on different biofunctions. Molecules were shown as nodes, and the biological relationship between two nodes was shown as an edge (line). All edges were supported by at least one reference from a textbook, the literature, or canonical information stored in the Ingenuity Pathway Knowledge Base (IPKB). Nodes with diverse shapes represented the different functional classes of gene products.

The networks were sorted depending on the score evaluated by the IPA, representing the significance of the molecules for the network. In addition, the IPA determined the significance of the associations between the focus molecules and canonical pathways using Fisher's exact test. To study the mechanism of EZP and MTX treatment of RA, canonical pathway analysis in IPA was accomplished by using the comparison module.

### Induction of Arthritis and Treatment

A total of 50 male Sprague-Dawley (SD) rats were purchased from the Research Institute of Experimental Animals, Chinese Academy of Medical Science (Animal license number: SCXK (Beijing) 2014-0013) at six weeks of age. Rats were maintained in the Experimental Animal Center of the Institute of Clinical Medical Sciences, China-Japan Friendship Hospital [Experiment Animal Center license number: SCXK (Beijing) 2016-0043]. Animals were kept in a specific pathogen-free environment with a temperature of 23°C (± 2°C) and a 12-hour alternating light/dark cycle. They had free access to standard rodent chow and water. All experimental procedures were approved and directed by the Institute of Clinical Medical Sciences, China-Japan Friendship Hospital, Beijing, China (No, 180111).

**Figure 1:**
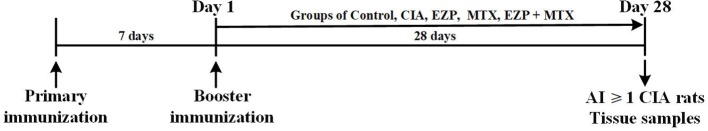
Experimental schedule. Male SD rats were immunized twice by intradermal injection with 50 µL bovine type II collagen emulsified with IFA on 7 days before day 1 and day 1. On day 1, the rats were randomly assigned into groups CIA, EZP, MTX, and EZP + MTX and given intragastric administration for 28 days. Tissue samples of the control rats and rats with AI ≥ 1 in the model were collected at the end-point of the experiment (day 28).

After three days of acclimation, 40 rats were injected intradermally with 50 µL of emulsified mixture consisting of bovine type II collagen (Chondrex, Inc., Redmond, WA, USA) and isopycnic incomplete Freund's adjuvant (IFA, Chondrex) at the tail root. Meanwhile, the remaining 10 rats were injected with the same volume of saline. Seven days after the initial immunization, the same method was used for booster immunizations. On the day of booster immunization, the rats that had received immunizations were randomly divided into four groups and treated by gavage: the collagen-induced arthritis group (CIA) received 10 mL of purified water per kg of weight, twice per day at 9:00 AM and 3:00 PM respectively; the MTX (Shanghai Sine Pharmaceutical Co., Ltd, Shanghai, China) treatment group (MTX) received 1.5 mg of MTX per kg of weight at 3:00 PM and 10 mL of purified water per kg of weight at 9:00 AM, twice per week, and for the other day, they received 10 mL of purified water per kg of weight, twice per day at 9:00 AM and 3:00 PM respectively; the Erzhi Pill (Yaodu Zhangshu Pharmaceutical Co., Ltd, Jiangxi, China; quality control showed in **Supplementary Material 1**) treatment group (EZP) received 1.8 g of Erzhi Pill per kg of weight, twice per day at 9:00 AM and 3:00 PM respectively; the Erzhi Pill and MTX combined group (EZP + MTX) was treated with Erzhi Pill at the same administration frequencies, dosages, and times as those of the EZP group and treated with MTX at the same administration frequencies and dosages as those of the MTX group but at 2:30 PM. In addition, the 10 unmodeled rats served as a normal control group (Control) treated with 10 mL of purified water per kg of weight, twice per day at the same time as the CIA group. The scheme of the design of the study and animal grouping are shown in **Figure 1**.

### Arthritis Assessment of Hind Limbs

From the day of administration of the treatment, rats were evaluated for the degree of swelling in the hind limbs every three days. The severity of arthritis per hind leg was expressed as the arthritic index (AI) score on a scale of 0 to 4 points according to conventional criteria (Zhao et al., 2018). Briefly, 0 = no change, 1 = redness or slight swelling, 2 = mild swelling, 3 = pronounced swelling, and 4 = deformity of and inability to use limb. Any modeling rats with an AI score of 0 at the last time point were deemed a failure of modeling and were excluded in the follow-up index.

### Histological Analysis of Ankle Joints

Rats were sacrificed after 28 days of treatment. The right hind ankle joints were fixed in formalin for seven days, followed by decalcification for one month. Then, the ankle joints were sectioned and stained with hematoxylin and eosin (H&E) under the guidance of a protocol. Histological score was evaluated by assessing inflammation, cartilage damage, bone damage, inflammatory cell infiltration, synovial hyperplasia, and pannus. The score was on a scale from 0 to 3 according to severity; the specific method was elaborated in an article by Zhao et al. (2018).

### Micro-Computed Tomography (Micro-CT) Analysis of Ankles and Paws

Micro-CT scans of the ankles and paws were performed to assess the extent of bone damage using a Skyscan1174 Micro CT (Bruker, Belgium). The matching software N-Recon was then used for 3D image reconstruction of both ankles and paws. Finally, 3D analysis of bone volume (BV), bone surface (BS), and BS/BV were performed *via* the matching software CT-AN.

### Serum Bone Metabolite Assessment by Enzyme-Linked Immunosorbent Assay (ELISA)

On the last day of treatment, blood was obtained from the abdominal aorta. After centrifugation at 3000 rpm for 10 minutes at 4°C, the serum was collected and stored at -80°C. The activity and function of OCs and OBs were evaluated by the levels of tartrate-resistant acid phosphatase (TRACP) and bone alkaline phosphatases (BALP) in the serum. They were measured using ELISA according to the protocol. ELISA kits were purchased from Nanjing Jiancheng Bioengineering Institute (Nanjing, China).

### Culture and Treatments of OB-Like Cells With Medicated Serum

The rat OB-like UMR-106 cell line (National Infrastructure of Cell Line Resource, China) was purchased and cultured in high-glucose Dulbecco's modified Eagle's medium (DMEM-H; HyClone, Logan, USA) containing 10% fetal bovine serum (FBS; Gibco BRL, Grand Island, NY, USA) in a constant-temperature incubator at 37°C, maintaining a moderate level of 5% CO_2_ and 95% saturation humidity. When the cells became 80-90% confluent, they were digested and inoculated for serial passage. UMR-106 cells in the logarithmic growth phase were reseeded in 96- and 24-well culture plates at densities of 1 × 10^5^ and 5 × 10^5^ cells/well, respectively; 24 hours later, the cells were used in follow-up experiments.

Once the dosages of EZP and MTX given to the rats had reached the maximum, the rat serum collected one hour after the last gavage was considered medicated serum following inactivation and filtration. UMR-106 cells seeded in cell plates were divided into five groups: the control serology group (Control), CIA serology group (CIA), MTX serology group (MTX), EZP serology group (EZP), and MTX+EZP serology group (MTX+EZP). UMR-106 cells were treated for 48 hours with culture medium supplemented with 15% medicated serum.

### Detection of OB-Like Cell Proliferation Rate *via* Cell Counting Kit-8 (CCK8) Assay

After 48 hours of intervention with medicated serum, the medium of UMR-106 cells was changed to DMEM-H containing 10% CCK8 (Dojindo, Japan). After reacting for 1.5 hours in the incubator, the absorbance was detected at wavelengths of 450 mm and 620 mm, and the cell proliferation rate was calculated.

### Evaluation of Relative Gene Expression of Wnt1/LRP5/*β*-Catenin Signaling Pathway Through Real-Time PCR

The left hindlimbs of rats were rapidly frozen with liquid nitrogen after being severed in the middle of the femur and removing the fur and muscles and then transferred to -80°C. Within a month, the bones were smashed in liquid nitrogen, and total RNA was extracted using the TRIzol reagent (Invitrogen, Carlsbad, CA, USA). Similarly, RNA was extracted from UMR-106 cells after 48 hours of intervention with medicated serum. The purity and quality of the isolated RNA were then examined with a NanoDrop One (Thermo Fisher Scientific, Waltham, MA, USA). gDNA was eliminated with gDNA Eraser, and the RNA was reverse transcribed with the PrimeScript RT reagent Kit (TaKaRa, Tokyo, Japan). The mRNA levels of selected genes were detected using SYBR Premix Ex Taq (TliRNaseH Plus) (TaKaRa) with the Quant Studio 5 Real-Time PCR System (Thermo Fisher Scientific) according to the manufacturer's guidelines. The cycling parameters were as follows: 95°C for 15 seconds, followed by 40 cycles at 95°C for 5 seconds and 60°C for 34 seconds. The samples were analyzed in duplicate, and their relative expression levels of genes were determined by normalization to the expression level of GAPDH. The primers used are shown in **Supplementary Material 2**.

### Statistical Analysis

Statistical analysis was carried out using GraphPad 8.0 software. The results were expressed as the mean ± *SEM*. Differences among groups were estimated by one-way analysis of variance (ANOVA) and a posthoc Tukey's test. Differences were considered significant when *P* < 0.05.

## Results

### Shared Signaling Pathways of MTX, EZP, and RA From Network Pharmacological Analysis

**Figure 2:**
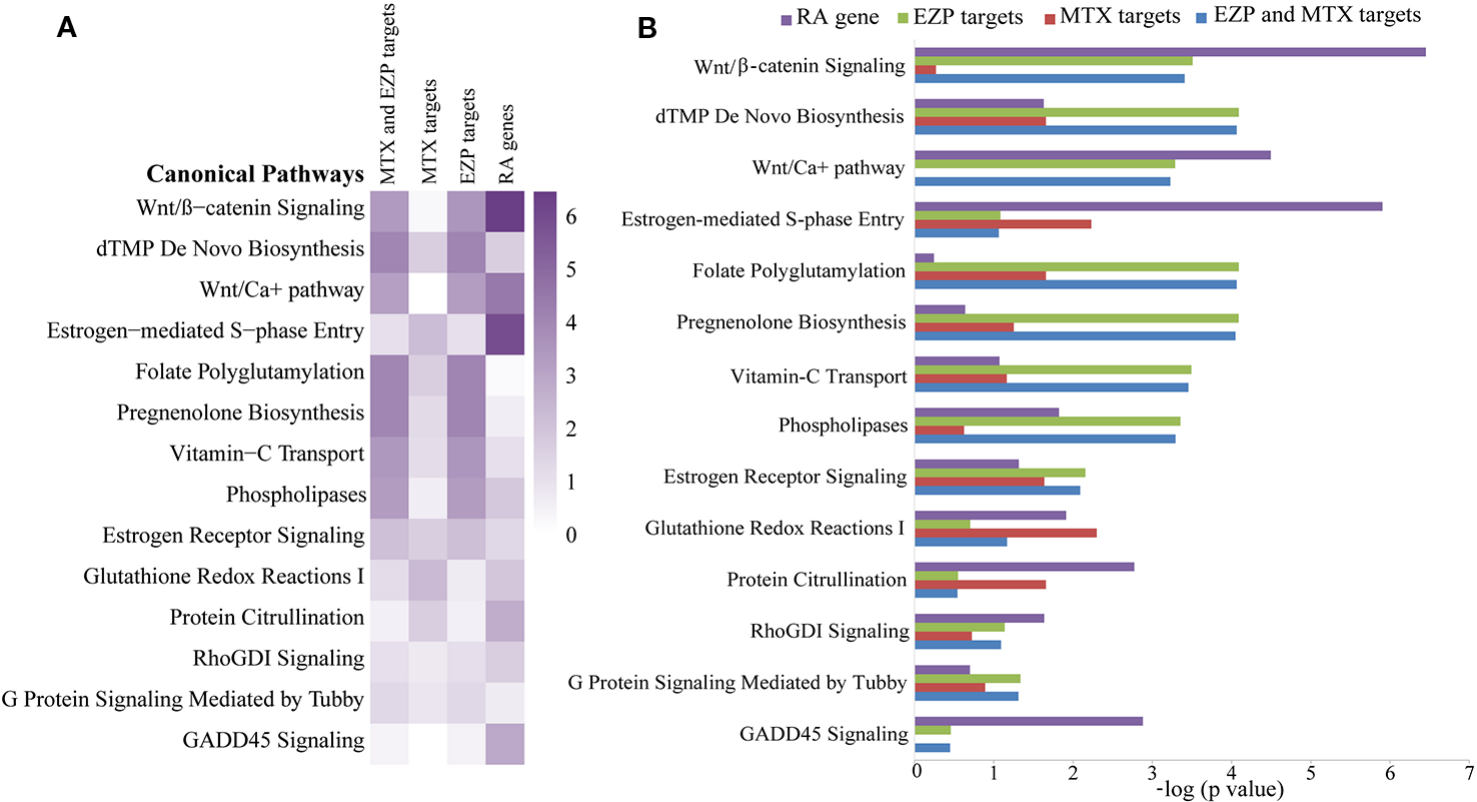
The results of bioinformatic analysis. Signaling pathways shared among the gene molecular networks related to rheumatoid arthritis (RA) and the protein target molecular networks of EZP and MTX in the context of cytokine and cellular immune signaling, identified by using the Ingenuity Pathway Analysis (IPA) compare module. The Wnt/*β*-catenin signaling pathway was the pathway focused on. **(A)** Heat map of the top 14 shared signaling pathways of EZP, MTX, and RA. **(B)** Bar graph of the top 14 shared signaling pathways of EZP, MTX, and RA.

A total of 41 chemical compounds (**Supplementary Material 3**) and 1454 targets (**Supplementary Material 4**) of EZP were obtained in this study. Meanwhile, 102 targets of MTX and 1148 RA-related genes were found (**Supplementary Materials 5** and **6**). In total, 328 signaling pathways of EZP and MTX were obtained, and 374 signaling pathways of RA were acquired. Among these signaling pathways, 297 were shared by EZP, MTX, and RA. The top 14 shared signaling pathways associated with cellular immune response and cytokine signaling are shown in **Figures 2A, B**. The Wnt/*β*-catenin signaling pathway was determined to be the top shared signaling pathway and the focus of further study.

### EZP Combined With MTX Ameliorated the Severity of Arthritis in CIA Rats

**Figure 3:**
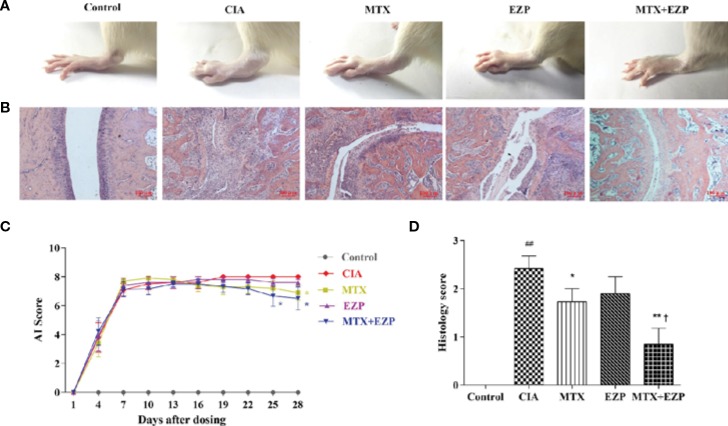
Effects of EZP combined with MTX on arthritis severity in CIA rats. **(A)** Representative images of the ankle joints after treatment. **(B)** Representative images of ankle pathology. **(C)** AI score of each group. **(D)** Histological score of each group. ^##^*P* < 0.01 compared with the control group; ^*^P < 0.05, ^**^P < 0.01 compared with the CIA group; ^†^*P* < 0.05 compared with the MTX group.

CIA rats were used in this study to verify the therapeutic effect of EZP and MTX on RA, and treatments were continued for 28 days. As shown in **Figure 3A**, ankle swelling was severe in CIA rats, and the three treatments were able to improve joint swelling to varying degrees. EZP + MTX had the best effect, while EZP alone had the weakest effect. The dynamic swelling scores are shown in **Figure 3C** and illustrate that, among all treatments, EZP + MTX produced the best results. From day 19 of treatment, all rats in the CIA group had the maximal AI score. Compared with the CIA group, the EZP group exhibited lower AI scores beginning on the 19th day of treatment, but there was no significant difference even at the end of the observation period. Although the MTX group had the highest score in the second week, MTX was effective after day 13, and a significant reduction in joint score was observed on the 28th day after treatment (*P* < 0.05). The therapeutic effects of EZP combined with MTX began to appear on the 7th day of treatment, and by the 25th day of treatment, the combination group had significantly reduced tumidness compared with the CIA group (*P* < 0.05). Although there was no significant difference between the MTX group and the EZP + MTX group, the AI score of the combined treatment group was significantly lower than that of the MTX group.

Pathological changes were shown in **Figures 3B, D**. The results suggested that the cartilage and bone were severely damaged in the CIA group, and this damage was accompanied by extensive synovial proliferation and inflammatory cell infiltration. EZP, MTX, and EZP+MTX treatments were able to reduce pathological injury to varying degrees. Histological score analysis showed that MTX and EZP + MTX prominently inhibited the histopathological changes in the ankle joints of CIA rats (*P* < 0.05; *P* < 0.01). These findings indicated that EZP alone was not effective in CIA rats but that it could coordinate with MTX to arrest the development and progression of CIA in rats.

### EZP Combined With MTX Showed Bone Protection in CIA Rats

**Figure 4:**
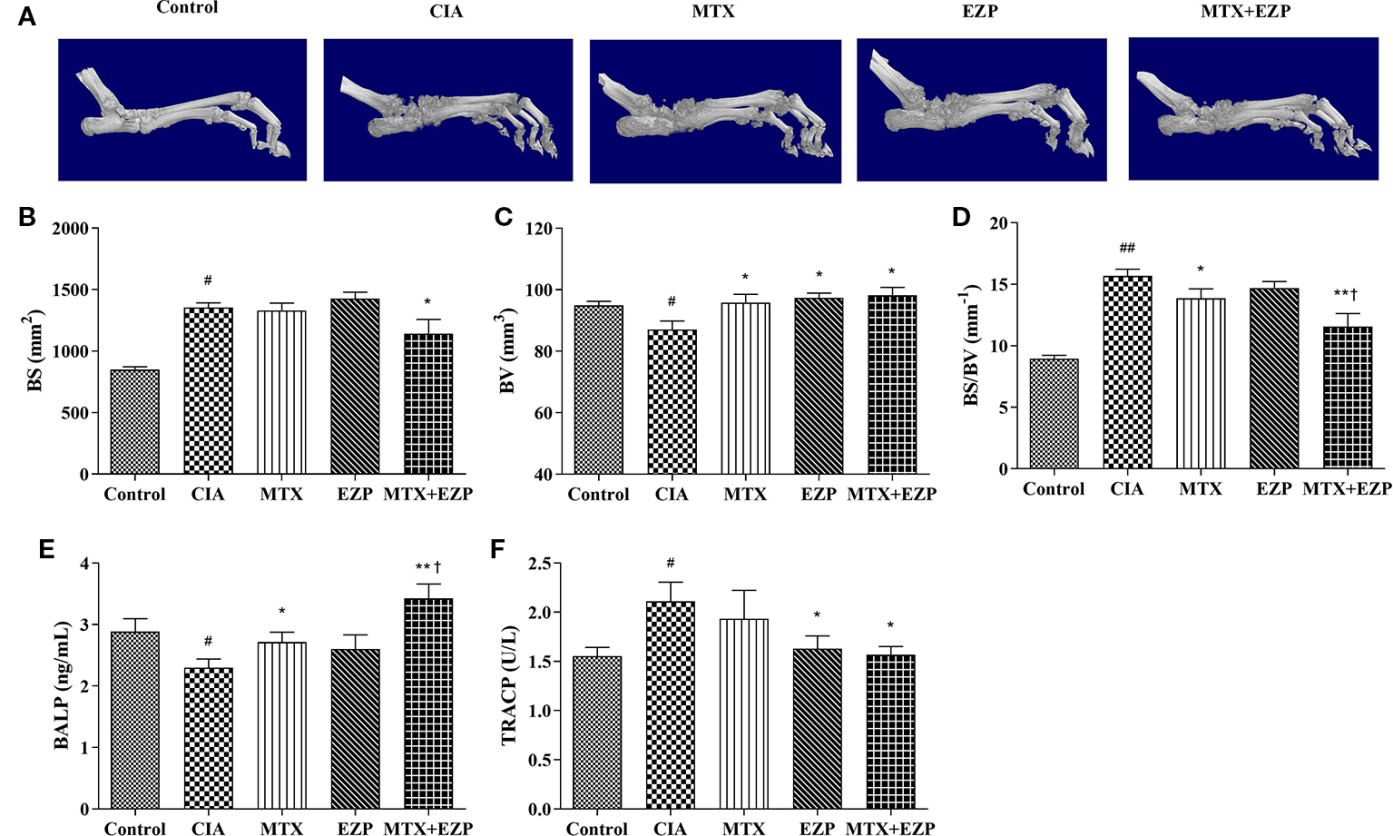
EZP and MTX synergistically reduced bone destruction in CIA rats. **(A)** Representative micro-CT images of ankle joints after treatment. **(B–D)** Line plots representing the BS, BV, and BS/BV values of all of the groups. **(E–F)** Bar plots representing the levels of BALP and TRACP in serum. ^#^*P* < 0.05, ^##^*P* < 0.01 compared with the control group; ^*^P < 0.05, ^**^P < 0.01 compared with the CIA group; ^†^*P* < 0.05 compared with the MTX group.

We then evaluated the therapeutic effects of EZP and MTX on the bone injury status *via* micro-CT (**Figure 4A**). The BV, BS, and BS/BV values in inflamed ankle joints were detected to quantify the extent of the bone remodeling in the different groups. The results showed that neither MTX nor EZP alone reduced the BS value, while the group treated with the combination of MTX and EZP exhibited a reduced BS compared with the CIA group (*P* < 0.05) (**Figure 4B**). BV was decreased in the CIA group, and all of the treatments increased it (*P* < 0.05) (**Figure 4C**). When considering the combination of BS and BV, we found that bone destruction was still present in the CIA group and that MTX and MTX + EZP played roles in bone protection (*P* < 0.05; *P* < 0.01) (**Figure 4D**). Interestingly, the combination of MTX and EZP was significantly better than MTX alone (*P* < 0.05). On the one hand, the micro-CT results preliminarily verified our prediction. On the other hand, the above results also suggested that the combination of the two drugs could inhibit bone absorption or increase osteogenesis.

In addition, the levels of BALP and TRACP in the serum were detected *via* ELISA to further verify the effect of EZP combined with MTX on bone metabolism. As shown in **Figure 4E**, the levels of BALP in CIA rats declined (*P* < 0.05), suggesting that modeling decreased OBs function. The levels of BALP were increased in the MTX group and EZP + MTX group (*P* < 0.05; *P* < 0.01), and those in the combination group were higher than those in the MTX group (*P* < 0.05), while there was no significant difference between the EZP and CIA groups. For TRACP (**Figure 4F**), CIA modeling increased the levels of TRACP in serum, and MTX, EZP, and MTX+EZP treatments could reduce these levels, but there was no difference between the EZP and MTX +EZP groups. The above results suggested that the effects of the combination of EZP and MTX were reflected in the protection of osteogenesis rather than effects on OCs.

### EZP Increased the Therapeutic Effects of MTX in CIA Rats Through the Wnt/*β*-Catenin Signaling Pathway

In order to further explore the molecular mechanism by which EZP enhanced the curative effects of MTX, we examined the Wnt/*β*-catenin signaling pathway and several key genes associated with the differentiation and maturation of OBs: Wnt1, LRP5, *β*-catenin, Runx2, bone gamma-carboxyglutamic-acid-containing proteins (BGP), and BALP.

**Figure 5:**
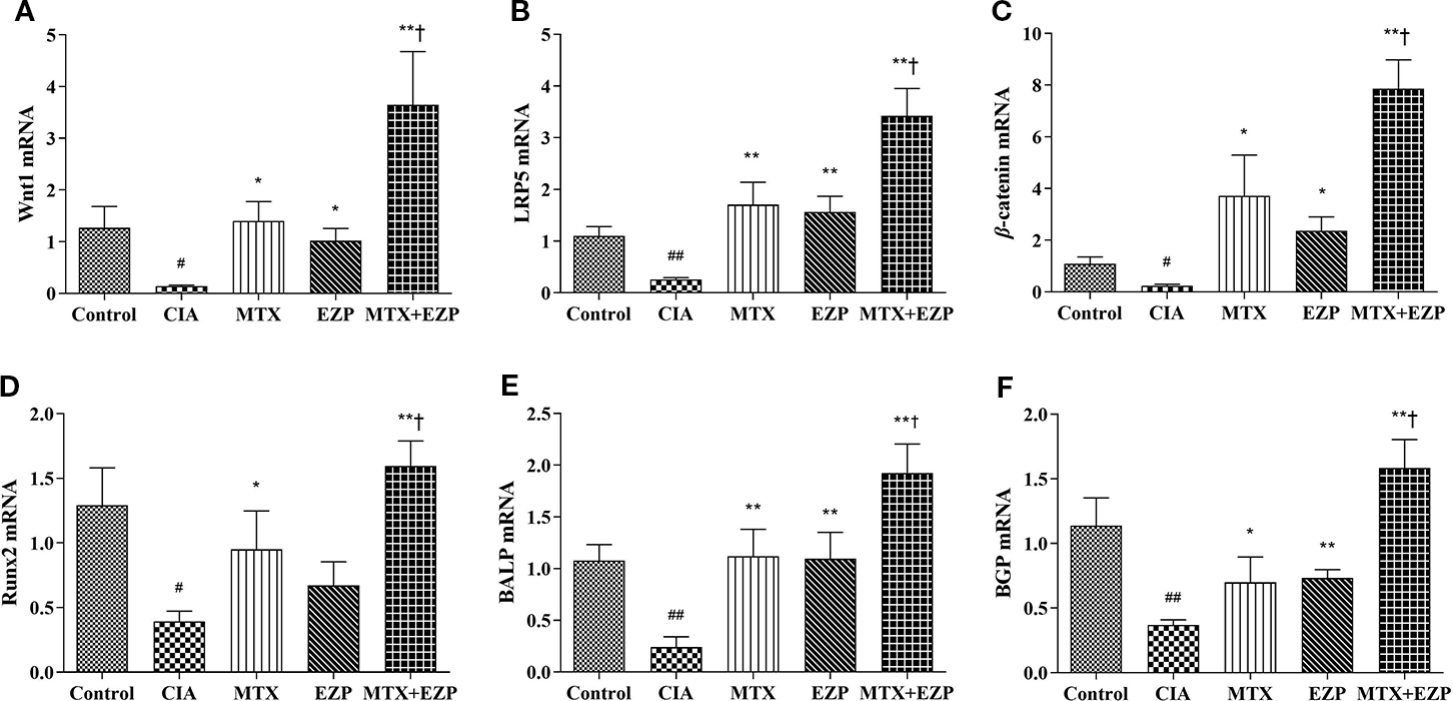
EZP and MTX synergistically promoted the differentiation and maturation of OBs in the CIA rat model. **(A–D)** Bar plots representing the relative mRNA expression levels of molecules related to the Wnt1/LRP5/*β*-catenin signaling pathway in the ankle joints. **(E–F)** RT-PCR results for BALP and BGP. ^#^*P* < 0.05, ^##^*P* < 0.01 compared with the control group; ^*^P < 0.05, ^**^P < 0.01 compared with the CIA group; ^†^*P* < 0.05 compared with the MTX group.

The effects of EZP and MTX on genes associated with osteogenesis in CIA joints are shown in **Figure 5**. As shown in **Figures 5A–D**, compared with that in the Control group, the gene expression of Wnt1, LRP5, *β*-catenin, and Runx2 in the CIA group was significantly downregulated (*P* < 0.05; *P* < 0.01); after EZP or MTX treatment alone or combined, except Runx2 in the EZP group, the gene expressions of Wnt1, LRP5, and *β*-catenin in the other two groups were significantly upregulated, and the combined treatment was more effective than MTX alone (*P* < 0.05; *P* < 0.01). As markers of OBs maturity, similar to the Wnt/*β*-catenin signaling pathway, BALP and BGP were observed to have significantly lower gene expression in the CIA group than in the Control group (*P* < 0.01); EZP, MTX, and MTX + EZP could increase the mRNA levels of both BALP and BGP to varying degrees, and the mRNA levels of BALP and BGP were increased more in the MTX + EZP group (*P* < 0.05) (**Figures 5E, F**). The results above showed that EZP and MTX promoted the differentiation and maturation of OBs. Moreover, EZP had a significant synergistic effect with MTX on OBs, mediated *via* the Wnt1/LRP5/*β*-catenin signaling pathway.

### Synergistic Effects of EZP Combined With MTX on OBs Mediated *via* the Wnt1/LRP5/*β*-Catenin Signaling Pathway

**Figure 6:**
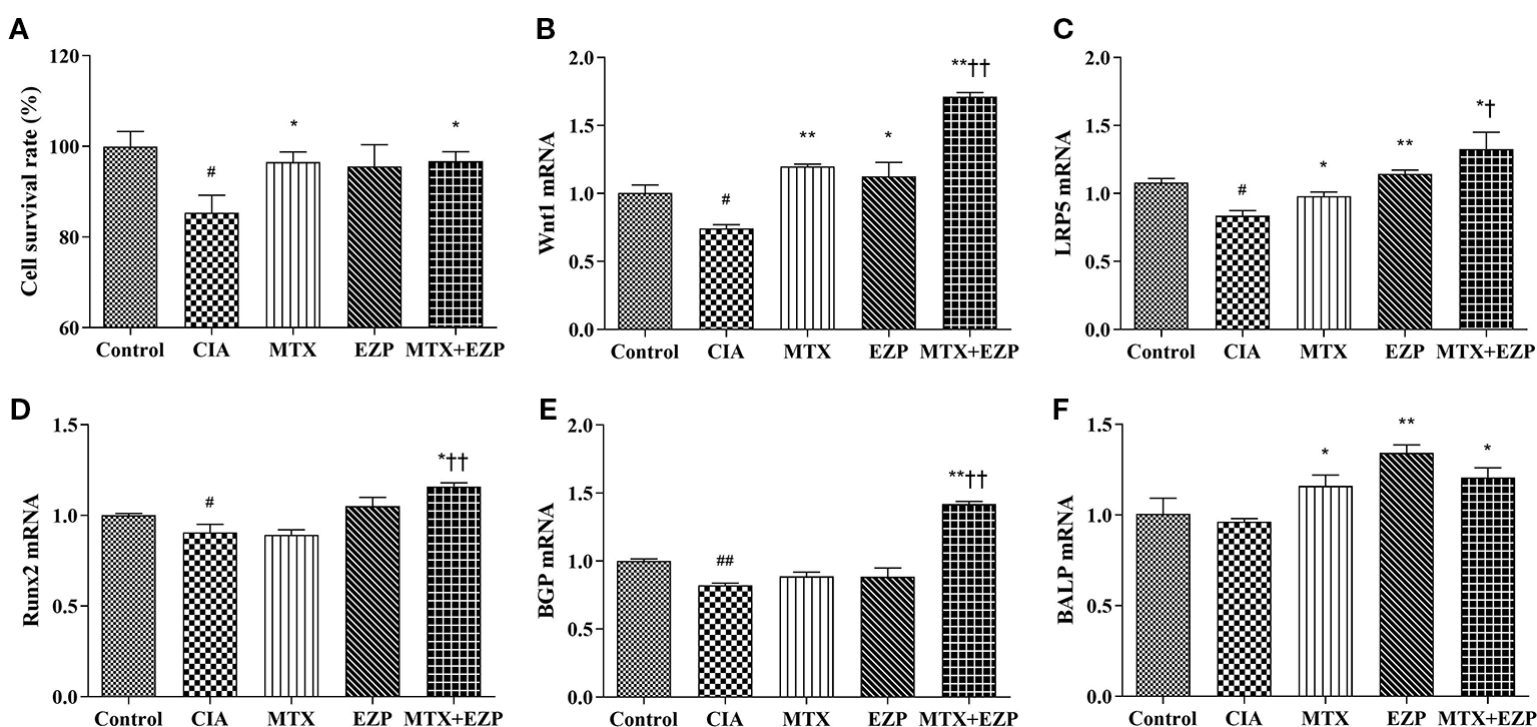
EZP and MTX synergistically promoted the differentiation and maturation of OBs. **(A)** Bar plots of the cell proliferation rate of each group. **(B–F)** Relative mRNA expression levels in OBs. ^#^*P* < 0.05, ^##^*P* < 0.01 compared with the control group; ^*^P < 0.05, ^**^P < 0.01 compared with the CIA group; ^†^*P* < 0.05, ^††^*P* < 0.01 compared with the MTX group.

Furthermore, we verified the results of our network pharmacology and animal experiments through cell experiments. OB-based results (**Figure 6**) showed that EZP could cooperate with MTX to promote OBs proliferation, differentiation, and maturation through the Wnt1/LRP5/*β*-catenin signaling pathway. **Figure 6A** showed that serum of CIA rats inhibited the survival of OBs (*P* < 0.05) and that MTX serum and MTX + EZP serum promoted cell proliferation compared with CIA serum (*P* < 0.05); however, there was no difference between MTX serum and MTX+EZP serum treatments on cell proliferation rates (*P >* 0.05). The results for Wnt1 and LRP5 expression in OBs were similar to the results from the joints; that is, the gene expressions were reduced in the CIA group, while all treatment groups restored them, and EZP + MTX was more effective than MTX (*P* < 0.05) (**Figures 6B, C**). The results for BGP and Runx2 were interesting. Neither MTX nor EZP alone altered the CIA-induced gene expression reduction, but the combination of MTX and EZP significantly increased the mRNA levels of both molecules (*P* < 0.05; *P* < 0.01) (**Figures 6D, E**). For BALP, modeling did not seem to alter gene expression, but all three treatment groups exhibited increased BALP gene levels to varying degrees (*P* < 0.05) (**Figure 6F**). These results bear out the results of the animal experiments showing that EZP had a significant synergistic effect with MTX on OBs that was mediated *via* the Wnt1/LRP5/*β*-catenin signaling pathway.

## Discussion

The RA management recommendations updated by the European League Against Rheumatism (EULAR) address conventional synthetic (cs) disease-modifying antirheumatic drugs (DMARDs), glucocorticoids (GC), biological (b) DMARDs, and targeted synthetic (ts) DMARDs (Smolen et al., 2017). However, the clinical use of these drugs is limited by adverse effects (Bijlsma and Buttgereit, 2016; Tarp et al., 2017; Wang et al., 2018), drug resistance (Peres et al., 2015), or high cost (Wu et al., 2014; Khilfeh et al., 2019), and none of these drugs directly target bone homeostasis. After treatment with MTX, approximately 30% of patients have no response, and 30% have side effects. Despite all this, because of its reasonable cost and remarkable curative effect, MTX has been the cornerstone drug for the treatment of RA for decades. For the purpose of increasing therapeutic effects or reducing toxicity, MTX has been used in the treatment of RA either as a single agent or in combination (Hazlewood et al., 2016).

From a clinical point of view, Chinese herbal formulas have good clinical efficacies in the treatment of RA (Huang et al., 2015), and some of them target the reestablishment of bone homeostasis in RA (Zhao et al., 2017; Zhao et al., 2018). Chinese herbal formulas have advantages that DMARDs lack, such as multiple targets, good curative effects, few adverse reactions, and low cost. EZP has exhibited a good effect in treating OP, and its effect mainly relies on bone protection (Cheng et al., 2011; Rufus et al., 2013; Sun et al., 2014). Given the unique advantages of EZP and MTX and the abilities of both agents to regulate bone homeostasis, we expect to enhance the effect of MTX by combining the two agents.

MTX combined with EZP for RA treatment involves multicomponent, multitarget, and multipathway effects from a global synergistic perspective. Traditional research methods have difficulty fully revealing the pharmacodynamics and corresponding mechanism of this combination. In recent years, along with the rapid progress in bioinformatics, network pharmacology has emerged as a holistic and efficient tool to decode the underlying mechanisms of multitarget treatments by analyzing various networks of complex and multilevel interactions (Zhang et al., 2015; Zhang et al., 2016; Zheng et al., 2016). Successful attempts to study pharmacodynamics have been achieved previously in our group by network pharmacology (Li et al., 2012; Zhao et al., 2015). In this study, based on a network pharmacological approach, the Wnt/*β*-catenin signaling pathway was the top signaling pathway shared by MTX, EZP, and RA, which meant that further study may focus on this pathway.

The CIA model is a valid experimental model for RA because the clinical changes observed are similar to those in RA patients (Alabarse et al., 2018). Furthermore, the pathological and biochemical changes in the bone and cartilage are similar to those in clinical patients (Daans et al., 2008). The basic pathological changes of RA as well CIA are chronic inflammation in the synovium, pannus formation, and progressive destruction of the articular cartilage and bone, leading to joint deformity and functional loss (Kato et al., 2015; Abasolo et al., 2019). Our ankle histological score and micro-CT results also showed that EZP cooperated with MTX to reduce cartilage and bone destruction and synovial cell and pannus proliferation, which means that EZP combined with MTX has effects on bone remodeling in RA.

Maintenance of bone homeostasis requires an exquisite balance between bone resorption by OCs and bone formation by OBs. BALP, a marker of OB differentiation and maturation from bone precursors, promotes extracellular mineralization *via* the release of inorganic phosphate (Halling et al., 2017). TRACP, a marker of OC differentiation and maturation, exerts phosphatase activity to phosphorylate osteopontin to inhibit the formation and growth of hydroxyapatite crystals (Halling et al., 2017). Our ELISA results showed that single-agent EZP had no effect on the levels of BALP but that the level of BALP increased more when EZP was combined with MTX than when MTX was used alone. The results were similar to those reported in the studies of Davies et al. (Davies et al., 2002; Davies et al., 2003). However, single-agent MTX did not decrease the levels of TRACP, and the combination of the two agents did not show an inhibitory effect on TRACP. These results suggest that EZP combined with MTX mainly acts on osteogenesis rather than osteoclasia.

For decades, studies have indicated that Wnt signaling is a critical regulator for stimulating and maintaining OB differentiation and activity in both humans and animals (Le Henaff et al., 2015; Sindhavajiva et al., 2018). When bound to an Fzd receptor complex, Wnt phosphorylates the Lrp coreceptors and recruits and coheres GSK-3*β* and Axin to the ligand-receptor complex. This complex results in the accumulation of cytoplasmic *β*-catenin. Finally, *β*-catenin translocates into the nucleus and induces the expression of target genes, such as Runx2. In OB differentiation, Wnt1 (Laine et al., 2013), Wnt3a (Li et al., 2019), and Wnt5a (Wang et al., 2019) play dominant roles in activating *β*-catenin through the binding receptor LRP5 (Gong et al., 2001; Chang et al., 2014) with other molecules, thereby promoting the transcription of the differentiation factor Runx2 (Jiang et al., 2017). In this study, both EZP alone and MTX alone increased the gene expression of Wnt1, LRP5, and *β*-catenin, indicating that both agents target the Wnt1/LRP5/*β*-catenin pathway. The combination of the two agents resulted in a significant additional induction of gene expression related to Wnt1/LRP5/*β*-catenin signaling, including Runx2, a gene that was not affected by the use of EZP. Furthermore, the results for BGP and BALP, two bone turnover markers, were consistent with those for the Wnt pathway. The results above preliminarily verified that the bone protection mechanism underlying the synergistic effect of EZP and MTX on RA was realized through the Wnt1/LRP5/*β*-catenin signaling pathway. In addition, a previous study from Sun et al. found that the anti-OP effect of EZP occurred through Wnt3a/LRP5/*β*-catenin signaling (Sun et al., 2014). We detected the gene expression of Wnt3a as well, but its level was too low for statistical analysis. The difference might be caused by the difference in bone tissues.

We further confirmed the mechanism underlying the synergistic effect of EZP and MTX on RA *in vitro* using the OB-like UMR-106 cell line. The results suggested that MTX and EZP-containing serum did not increase the proliferation rate of UMR-106 cells, indicating that the treatment of RA by EZP and MTX was not associated with promoting the proliferation of OBs. The gene expression results for the Wnt1/LRP5/*β*-catenin signaling pathway finally determined that the synergistic effects of EZP combined with MTX were achieved by increasing OB differentiation *via* the Wnt1/LRP5/*β*-catenin signaling pathway.

In conclusion, this study explored the anti-RA effect of EZP combined with MTX through network pharmacological analysis, a CIA rat model, and cell experiments. Our results demonstrated that EZP might exert a synergistic effect in combination with MTX to regulate OBs in RA through the Wnt1/LRP5/*β*-catenin signaling pathway.

## Data Availability Statement

The datasets generated for this study are available on request to the corresponding author.

## Ethics Statement

All experimental procedures were approved and directed by the Institute of Clinical Medical Sciences, China-Japan Friendship Hospital, Beijing, China (No: 180111).

## Author Contributions

CX, XyL, and XcL designed the conceptual framework of the study. DF and XcL designed experiments and wrote the paper. LL, CL, YT, YX, MF, and HZ performed experiments. YT, XcL, and DF analyzed the data.

## Conflict of Interest

The authors declare that the research was conducted in the absence of any commercial or financial relationships that could be construed as a potential conflict of interest.

The reviewer JC declared a shared affiliation, with no collaboration, with the authors, XL, YX, to the handling editor at time of review.
