# Supplementary material for: Synergistic Effects of Erzhi Pill Combined With Methotrexate on Osteoblasts Mediated via the Wnt1/LRP5/β-Catenin Signaling Pathway in Collagen-Induced Arthritis Rats
Source: Front Pharmacol. 2020 Mar 11;11:228. doi: 10.3389/fphar.2020.00228 (PMC7079734; doi:10.3389/fphar.2020.00228)
Supplement: Supplementary file 1 [file DataSheet_1.docx]

Supplementary Material

**Quality control of five compounds from Erzhi Pill using HPLC-DAD/UV have been added in the revised manuscript. The detailed revisions are listed as following.**

1. **Introduction**

Erzhi Pill (EZP), recorded in the Chinese Pharmacopoeia (Chinese Pharmacopoeia Commission, 2015), is a classical Chinese patent medicine consisted of *Ligustrum lucidum Ait*. and *Eclipta prostrata L.* and plays the role of against liver injury and antiosteoporotic effect ^1, 2^. So far, more than 100 compounds ^3^ and 88 compounds ^4^ have been identified in *Ligustrum lucidum Ait*. and *Eclipta prostrata L* respectively.

As a Chinese Materia Medica standardized product approved by China National Medical Products Administration (NMPA), the quality control of EZP complied with the standards in Chinese Pharmacopoeia (total content of specnuezhenide were not less than 4 mg/g by HPLC) ^[6]^. Furthermore, in order to comprehensively guarantee the quality of EZP, five characteristic and bioactive components have been selected as quality control markers from the involved two *herbs* ^[5]^. They are specnuezhenide, salidroside and tyrosol from *Ligustrum lucidum Ait*., wedelolactone from *Eclipta prostrata L.* and luteolin from both *herbs.*

In this study, we still use the five components to evaluate the quality of EZP used in our experimental and the structures of the five compounds were shown in **Supplementary Figure 1**.The methodology had been verified in reference 5 and the results indicated that the quality of EZP used in this study exceeded the requirements of Chinese Pharmacopoeia.


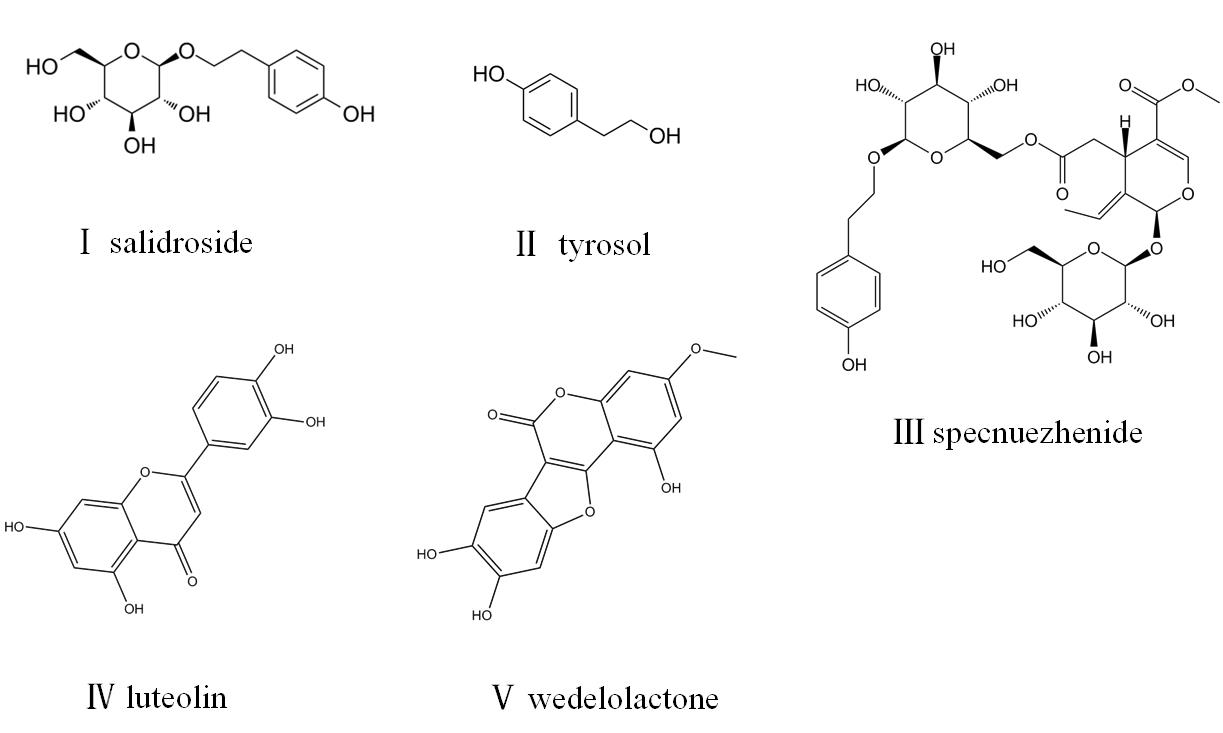


**Supplementary Figure 1.** The structures of the five compounds detected in EZP. Ⅰ: salidroside, Ⅱ: tyrosol, Ⅲ: specnuezhenide, Ⅳ: luteolin, Ⅴ: wedelolactone.

1. **Materials and methods**

**2.1 Chemicals**

An HPLC analysis was performed by Shimadzu LC-20A System (Shimadzu, Japan). The water was high pure water, methanol and acetonitrile are HPLC-grade (Thermo Fisher Scientific Inc., Shanghai, China). The reference compounds specnuezhenide, salidroside, tyrosol, luteolin and wedelolactone (purity: 97.3%, 99.4%, 100%, 93.3%, 99.6%, respectively) were purchased from National Institutes for Food and Drug Control (Beijing, China).

**2.2 Sample preparation**

About 1-2 g powder of EZP was weighed precisely and added with 75% methanol to a 25 mL Erlenmeyer flask, and the flask was accurately weighed. The sample was followed by heat reflux for 1 hour and weighed again. Then, any solvent lost was added after being cooled to room temperature. Subsequently, the mixture was filtered through a 0.45 μm membrane filter. Finally, an appropriate amount of specnuezhenide, salidroside, tyrosol, luteolin and wedelolactone was taken and precisely weighed. Methanol was added to make a mixed control solution with mass concentration of 250mg/L, 200mg/L, 80mg/L, 2mg/L and 2mg/L, respectively and stored at 4°C for later analysis.

**2.3 Chromatographic conditions**

The chromatographic column was used Phenomenex Gemini C_18_ (4.6 mm×250 mm, 5 μm). The mobile phase was composed of solvent A (acetonitrile) and solvent B (0.2% formic acid-water 0.5%) with a gradient elution (0-15 min, 10%-30% A; 15-25 min, 30%-33% A; 25-30 min, 33% A). The flow rate of the mobile phase was 1 mL/min and the column temperature were maintained at 30°C. The injection volume was 10 µL. The detective wavelengths were at 225 nm (specnuezhenide, salidroside and tyrosol) and 349 nm (luteolin and wedelolactone).

**3. Results**

We tested the three batches of EZP used in our experiment. All the five analytes were successfully separated using the HPLC method and the one with the shortest retention time was salidroside and the one with the longest retention time was wedelolactone (**Supplementary Figure 2**). According to the established methodology ^[5]^, these five selected compounds were quantitatively determined. The results showed that the relatively contents of five compounds were stable among three batches and the content of specnuezhenide was up to 22.34mg/g (**Supplementary Table 1**), which are satisfied or exceeded the requirements of Chinese Pharmacopoeia.


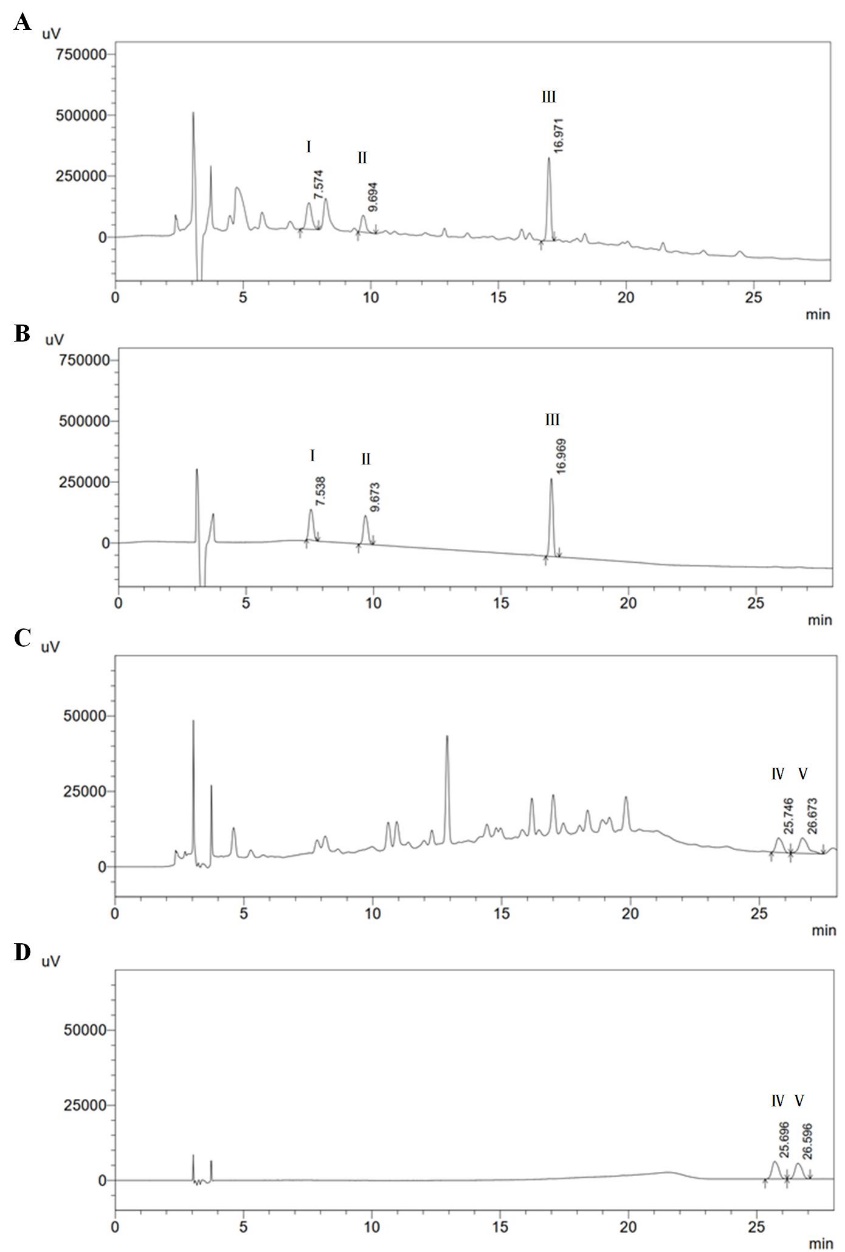


**Supplementary Figure 2.** The chromatography of determination of 5 components in EZP by UPLC. (A) and (C) were samples, (B) and (D) were reference compounds; (A)and (B) were at 225nm, (C) and (D) were at 349 nm; Ⅰ: salidroside, Ⅱ: tyrosol, Ⅲ: specnuezhenide, Ⅳ: luteolin, Ⅴ: wedelolactone.

**Supplementary Table 1.** Determination of five components in EZP samples ($\bar{x}$, n=2, mg/g)

|  | specnuezhenide | salidroside | tyrosol | luteolin | wedelolactone |
| --- | --- | --- | --- | --- | --- |
| Batch 1 | 22.36 | 5.61 | 1.50 | 0.604 | 0.301 |
| Batch 2 | 22.37 | 6.00 | 1.49 | 0.616 | 0.319 |
| Batch 3 | 22.34 | 5.47 | 1.49 | 0.588 | 0.305 |

**References**

1. Zhao HM, Zhang XY, Lu XY, Yu SR, Wang X, Zou Y et al. Erzhi Pill((R)) Protected Experimental Liver Injury Against Apoptosis via the PI3K/Akt/Raptor/Rictor Pathway. Front Pharmacol (2018) 9:283. doi: 10.3389/fphar.2018.00283.

2. Yao W, Gu H, Zhu J, Barding G, Cheng H, Bao B et al. Integrated plasma and urine metabolomics coupled with HPLC/QTOF-MS and chemometric analysis on potential biomarkers in liver injury and hepatoprotective effects of Er-Zhi-Wan. Anal Bioanal Chem (2014) 406:7367-78. doi: 10.1007/s00216-014-8169-x.

3. Gao L, Li C, Wang Z, Liu X, You Y, Wei H et al. Ligustri lucidi fructus as a traditional Chinese medicine: a review of its phytochemistry and pharmacology. Nat Prod Res (2015) 29:493-510. doi: 10.1080/14786419.2014.954114.

4. Feng L, Zhai YY, Xu J, Yao WF, Cao YD, Cheng FF et al. A review on traditional uses, phytochemistry and pharmacology of Eclipta prostrata (L.) L. J Ethnopharmacol (2019) 245:112109. doi: 10.1016/j.jep.2019.112109

5. Fan MX, Tan C, Wang MM, Du XW, Liu Y, Wang JH et al. Comprehensive evaluation of quality of Erzhiwan based on five characteristic components. Chinese Journal of Experimental Traditional Medical Formulae (article in Chinese) (2018) 24:88-92. doi: 10.13422/j.cnki. Syfjx.20181607.

6. Committee for the pharmacopoeia of China, Pharmacopoeia of China, Part I; China Medical Science and Technology Press, Beijing, China, (2015): 437.
