# Supplementary material for: Synergistic Effects of Erzhi Pill Combined With Methotrexate on Osteoblasts Mediated via the Wnt1/LRP5/β-Catenin Signaling Pathway in Collagen-Induced Arthritis Rats
Source: Front Pharmacol. 2020 Mar 11;11:228. doi: 10.3389/fphar.2020.00228 (PMC7079734; doi:10.3389/fphar.2020.00228)
Supplement: Supplementary file 2 [file Table_1.docx]

**Supplementary Table 1.** Determination of five components in EZP samples ($\bar{x}$, n=2, mg/g)

|  | specnuezhenide | salidroside | tyrosol | luteolin | wedelolactone |
| --- | --- | --- | --- | --- | --- |
| Batch 1 | 22.36 | 5.61 | 1.50 | 0.604 | 0.301 |
| Batch 2 | 22.37 | 6.00 | 1.49 | 0.616 | 0.319 |
| Batch 3 | 22.34 | 5.47 | 1.49 | 0.588 | 0.305 |

**References**

1. Zhao HM, Zhang XY, Lu XY, Yu SR, Wang X, Zou Y et al. Erzhi Pill((R)) Protected Experimental Liver Injury Against Apoptosis via the PI3K/Akt/Raptor/Rictor Pathway. Front Pharmacol (2018) 9:283. doi: 10.3389/fphar.2018.00283.

2. Yao W, Gu H, Zhu J, Barding G, Cheng H, Bao B et al. Integrated plasma and urine metabolomics coupled with HPLC/QTOF-MS and chemometric analysis on potential biomarkers in liver injury and hepatoprotective effects of Er-Zhi-Wan. Anal Bioanal Chem (2014) 406:7367-78. doi: 10.1007/s00216-014-8169-x.

3. Gao L, Li C, Wang Z, Liu X, You Y, Wei H et al. Ligustri lucidi fructus as a traditional Chinese medicine: a review of its phytochemistry and pharmacology. Nat Prod Res (2015) 29:493-510. doi: 10.1080/14786419.2014.954114.

4. Feng L, Zhai YY, Xu J, Yao WF, Cao YD, Cheng FF et al. A review on traditional uses, phytochemistry and pharmacology of Eclipta prostrata (L.) L. J Ethnopharmacol (2019) 245:112109. doi: 10.1016/j.jep.2019.112109

5. Fan MX, Tan C, Wang MM, Du XW, Liu Y, Wang JH et al. Comprehensive evaluation of quality of Erzhiwan based on five characteristic components. Chinese Journal of Experimental Traditional Medical Formulae (article in Chinese) (2018) 24:88-92. doi: 10.13422/j.cnki. Syfjx.20181607.

6. Committee for the pharmacopoeia of China, Pharmacopoeia of China, Part I; China Medical Science and Technology Press, Beijing, China, (2015): 437.
