# Supplementary material for: Synergistic Effects of Erzhi Pill Combined With Methotrexate on Osteoblasts Mediated via the Wnt1/LRP5/β-Catenin Signaling Pathway in Collagen-Induced Arthritis Rats
Source: Front Pharmacol. 2020 Mar 11;11:228. doi: 10.3389/fphar.2020.00228 (PMC7079734; doi:10.3389/fphar.2020.00228)
Supplement: Supplementary file 3 [file Table_2.docx]

Supplementary Material

# Supplementary Table 2. Primer sequences used for quantitative PCR

| Gene | Forward primer (5′-3′) | Reverse primer (5′-3′) |
| --- | --- | --- |
| Wnt1 | CTCCACGAACCTGCTGACAGATTC | CGGATCAGTCGTCGCTGCTTG |
| LRP5 | CTTCATCCACCGTGCCAACCTG | TCTGCCAGTCTGTCCAGTAGAGTG |
| β-catenin | ATATTGACGGGCAGTATGCA | TCAAACTGCGTGGATGGGAT |
| Runx2 | TCCGCCACCACTCACTACCAC | GGAACTGATAGGACGCTGACGAAG |
| BALP | CACGGCGTCCATGAGCAGAAC | CAGGCACAGTGGTCAAGGTTGG |
| BGP | ACCTTACTGCCCTCCTGCTTGG | GGACCCTCTCTCTGCTCACTCTG |
| GAPDH | TGCCAGCCTCGTCCCGTAGAC | CCTCACCCCATTTGATGTTAG |
