# Supplementary material for: Synergistic Effects of Erzhi Pill Combined With Methotrexate on Osteoblasts Mediated via the Wnt1/LRP5/β-Catenin Signaling Pathway in Collagen-Induced Arthritis Rats
Source: Front Pharmacol. 2020 Mar 11;11:228. doi: 10.3389/fphar.2020.00228 (PMC7079734; doi:10.3389/fphar.2020.00228)
Supplement: Supplementary file 4 [file Table_3.docx]

Supplementary Material

# Supplementary Table 3. The compounds of Erzhi Pill (EZP)

| Number | Compounds |
| --- | --- |
| 1 | 2-(Buta-1,3-diynyl)-5-(4-chloro-3-hydroxybut-1-ynyl) thiophene |
| 2 | alpha-Terithenyl acetate |
| 3 | alpha-Terthienyl methanol |
| 4 | Butein |
| 5 | Butin |
| 6 | Chloromaloside |
| 7 | Demethylwedelolactone-7-glucoside |
| 8 | Demissine |
| 9 | Ecliptasaponin B |
| 10 | Edulinine |
| 11 | Isodesacetyluvaricin |
| 12 | Niacin, Nicotinic Acid |
| 13 | 3,4-Dihydroxybenzoic Acid, Protocatechuic Acid |
| 14 | Testosterone |
| 15 | PHB |
| 16 | Acacini, Linarin |
| 17 | Acacetin |
| 18 | Hexahydrofarnesyl Acetone |
| 19 | Cinaroside |
| 20 | (1R,2R,4R)-Dihydrocarveol |
| 21 | alpha-T COOH deriv |
| 22 | 1,3,8,9-tetrahydroxybenzofurano[3,2-c] chromen-6-one |
| 23 | Ecliptasaponin D_qt |
| 24 | Ecliptasaponin |
| 25 | 3-[(2S)-2,3-dihydroxy-3-methyl-butyl]-4-methoxy-1-methyl-carbostyril |
| 26 | 3'-O-Methylorobol |
| 27 | TES |
| 28 | Β-Guaiene |
| 29 | Benzofurano(3',2':3,4)coumarin |
| 30 | (3S,8S,9S,10R,13R,14S,17R)-17-[(1R,4R)-1,4-dimethylhexyl]-10,13-dimethyl-2,3,4,7,8,9,11,12,14,15,16,17-dodecahydro-1H-cyclopenta[a]phenanthren-3-ol |
| 31 | (3S,8S,9S,10R,13R,14S,17R)-17-[(E,1R,4R)-1,4-dimethylhex-2-enyl]-10,13-dimethyl-2,3,4,7,8,9,11,12,14,15,16,17-dodecahydro-1H-cyclopenta[a]phenanthren-3-ol |
| 32 | Pratensein |
| 33 | Caulophyllogenin |
| 34 | (X {2212})-Nicotine |
| 35 | wedelolactone |
| 36 | Β-Amyrin |
| 37 | (-)-Caryophyllene Oxide, (1R,4R,6R,10S)-4,12,12-Trimethyl-9-Methylidene-5-Oxatricyclo [8.2.0.0~4,6~] Dodecane |
| 38 | Luteolin |
| 39 | Apigenin-7-O-Glucoside, Apigetrin, Cosmosiin |
| 40 | Apigenin |
| 41 | Guercetol, Quercetin, Quercetin, Sophoretin, Meletin, Xanthaurine |
