# Supplementary material for: Synergistic Effects of Erzhi Pill Combined With Methotrexate on Osteoblasts Mediated via the Wnt1/LRP5/β-Catenin Signaling Pathway in Collagen-Induced Arthritis Rats
Source: Front Pharmacol. 2020 Mar 11;11:228. doi: 10.3389/fphar.2020.00228 (PMC7079734; doi:10.3389/fphar.2020.00228)
Supplement: Supplementary file 5 [file Table_4.docx]

Supplementary Material

# Supplementary Table 4. The targets of Erzhi Pill (EZP)

| Number | Targets name |
| --- | --- |
| 1 | MSMO1 |
| 2 | SLC22A16 |
| 3 | CYP2C8 |
| 4 | SLC22A11 |
| 5 | RPL11 |
| 6 | AMT |
| 7 | AMN |
| 8 | CASK |
| 9 | GSS |
| 10 | ALKBH2 |
| 11 | GGH |
| 12 | SOAT2 |
| 13 | DPP4 |
| 14 | PYCRL |
| 15 | TOP2A |
| 16 | RAB8B |
| 17 | TOP2B |
| 18 | SHMT1 |
| 19 | COMTD1 |
| 20 | OTC |
| 21 | SHMT2 |
| 22 | FBP1 |
| 23 | RBP1 |
| 24 | RBP2 |
| 25 | F10 |
| 26 | RBP3 |
| 27 | GPR133 |
| 28 | IGF1R |
| 29 | RBP4 |
| 30 | TARS |
| 31 | LARS |
| 32 | RALBP1 |
| 33 | RBP5 |
| 34 | RBP7 |
| 35 | PLA2G1B |
| 36 | DHRS11 |
| 37 | PRKAB1 |
| 38 | SLC8A1 |
| 39 | FAXDC2 |
| 40 | PRKAB2 |
| 41 | CROT |
| 42 | UGDH |
| 43 | ALDH18A1 |
| 44 | EHHADH |
| 45 | GLUL |
| 46 | LCK |
| 47 | RAN |
| 48 | SESN2 |
| 49 | REN |
| 50 | IVD |
| 51 | NDUFS1 |
| 52 | EPHB2 |
| 53 | ZNF259 |
| 54 | SLC2A10 |
| 55 | NDUFS4 |
| 56 | NDUFS3 |
| 57 | LSS |
| 58 | COX6C |
| 59 | CDA |
| 60 | NDUFS2 |
| 61 | SLC2A11 |
| 62 | NDUFS5 |
| 63 | NDUFC1 |
| 64 | PDE2A |
| 65 | SLC2A12 |
| 66 | NDUFS6 |
| 67 | NDUFC2 |
| 68 | NDUFS7 |
| 69 | CREB1 |
| 70 | LCT |
| 71 | UQCRB |
| 72 | NDUFS8 |
| 73 | INS |
| 74 | AIFM1 |
| 75 | C8G |
| 76 | KHSRP |
| 77 | SC4MOL |
| 78 | DTYMK |
| 79 | UQCRH |
| 80 | HCN2 |
| 81 | RRM2B |
| 82 | QARS |
| 83 | YARS |
| 84 | NDUFB10 |
| 85 | OGFOD1 |
| 86 | ACSS1 |
| 87 | ARG1 |
| 88 | OGFOD2 |
| 89 | EEF1A1 |
| 90 | ACSS2 |
| 91 | ARG2 |
| 92 | PC |
| 93 | UQCRQ |
| 94 | MTHFR |
| 95 | CYP4B1 |
| 96 | TH |
| 97 | SHBG |
| 98 | CYP1A1 |
| 99 | CYP1A2 |
| 100 | EIF3F |
| 101 | CYP26B1 |
| 102 | IRGC |
| 103 | PTGIR |
| 104 | DLAT |
| 105 | PTGIS |
| 106 | GYG1 |
| 107 | RLBP1 |
| 108 | ICAM1 |
| 109 | CYC1 |
| 110 | TSTA3 |
| 111 | GRIN3A |
| 112 | GRIN3B |
| 113 | BDH1 |
| 114 | HSP90B1 |
| 115 | KCNJ1 |
| 116 | PNPO |
| 117 | ALAD |
| 118 | BAG1 |
| 119 | CPT2 |
| 120 | THBD |
| 121 | GPT2 |
| 122 | IL1B |
| 123 | ATP2A1 |
| 124 | GPD1 |
| 125 | DGUOK |
| 126 | KCNJ8 |
| 127 | ME1 |
| 128 | ABCB11 |
| 129 | ME2 |
| 130 | ME3 |
| 131 | WARS2 |
| 132 | BECN1 |
| 133 | SCN2A |
| 134 | SCN2B |
| 135 | RORA |
| 136 | MYC |
| 137 | RPS6KA3 |
| 138 | DAPK1 |
| 139 | WARS |
| 140 | RPS6KA4 |
| 141 | NCF1 |
| 142 | NCF2 |
| 143 | RCVRN |
| 144 | NCF4 |
| 145 | MME |
| 146 | ATOX1 |
| 147 | PRL |
| 148 | VDR |
| 149 | CDH5 |
| 150 | UQCRFS1 |
| 151 | MIF |
| 152 | PADI4 |
| 153 | PNP |
| 154 | GRIK1 |
| 155 | PVR |
| 156 | GRIK2 |
| 157 | SOAT1 |
| 158 | GPHN |
| 159 | GRIK3 |
| 160 | UGT2B15 |
| 161 | GRIK4 |
| 162 | DPH5 |
| 163 | GRIK5 |
| 164 | MUT |
| 165 | DBH |
| 166 | GSR |
| 167 | DBI |
| 168 | SORD |
| 169 | GCK |
| 170 | SETD7 |
| 171 | SLC25A10 |
| 172 | AMY1A |
| 173 | UCKL1 |
| 174 | ALKBH3 |
| 175 | SLC25A15 |
| 176 | SLC3A2 |
| 177 | SLCO3A1 |
| 178 | ADSSL1 |
| 179 | SLC25A18 |
| 180 | CDK15 |
| 181 | DDX6 |
| 182 | ENPP1 |
| 183 | SLC15A1 |
| 184 | TMLHE |
| 185 | HAO1 |
| 186 | HAO2 |
| 187 | PON3 |
| 188 | SLC6A14 |
| 189 | PROC |
| 190 | PCMT1 |
| 191 | ACVR1B |
| 192 | PI4K2B |
| 193 | WEE1 |
| 194 | COX6B1 |
| 195 | SLC6A15 |
| 196 | PROS1 |
| 197 | ACTA1 |
| 198 | CASR |
| 199 | SLC22A10 |
| 200 | TUBG1 |
| 201 | RPL15 |
| 202 | SLC6A19 |
| 203 | ADRA2A |
| 204 | LCN1 |
| 205 | ADRA2B |
| 206 | TCN1 |
| 207 | CYP2C9 |
| 208 | MYCN |
| 209 | PROSC |
| 210 | ADRA2C |
| 211 | LCN2 |
| 212 | TCN2 |
| 213 | RPL19 |
| 214 | MDH1 |
| 215 | IDH1 |
| 216 | IDH2 |
| 217 | MDH2 |
| 218 | ADH4 |
| 219 | SLC25A2 |
| 220 | SFTPD |
| 221 | ADH5 |
| 222 | ADH6 |
| 223 | SLC25A4 |
| 224 | ADH7 |
| 225 | NISCH |
| 226 | ALDH6A1 |
| 227 | AKR1B1 |
| 228 | BGLAP |
| 229 | RPL3 |
| 230 | PROZ |
| 231 | P2RY2 |
| 232 | RPL8 |
| 233 | SLC7A11 |
| 234 | IKBKB |
| 235 | PROCR |
| 236 | REG1A |
| 237 | MAOA |
| 238 | HSD11B1 |
| 239 | PLAT |
| 240 | MAOB |
| 241 | HSD11B2 |
| 242 | CTSB |
| 243 | CTSF |
| 244 | ITPR1 |
| 245 | MGAM |
| 246 | BIRC5 |
| 247 | JDP2 |
| 248 | P4HTM |
| 249 | HNF4A |
| 250 | ALOX15 |
| 251 | CACNB1 |
| 252 | CACNB2 |
| 253 | PAPOLA |
| 254 | CACNB3 |
| 255 | RDH5 |
| 256 | JAK1 |
| 257 | CHRM1 |
| 258 | HPRT1 |
| 259 | CHRM2 |
| 260 | CACNB4 |
| 261 | HNF4G |
| 262 | TYR |
| 263 | RAC1 |
| 264 | RAC2 |
| 265 | RDH8 |
| 266 | AASS |
| 267 | CHRM3 |
| 268 | CHRM4 |
| 269 | TAT |
| 270 | CHKA |
| 271 | CHRM5 |
| 272 | CHKB |
| 273 | CLEC4E |
| 274 | SULT2B1 |
| 275 | CLEC14A |
| 276 | HSP90AA1 |
| 277 | SUCLA2 |
| 278 | SULT2B1 |
| 279 | FURIN |
| 280 | ADH1A |
| 281 | ADH1B |
| 282 | GNMT |
| 283 | AGXT2 |
| 284 | PLEKHA1 |
| 285 | GABBR1 |
| 286 | FDXR |
| 287 | UQCRC1 |
| 288 | OAS1 |
| 289 | SLCO1B1 |
| 290 | SERPINA5 |
| 291 | GABBR2 |
| 292 | SERPINA1 |
| 293 | UQCRC2 |
| 294 | PPT1 |
| 295 | PLEKHA4 |
| 296 | SLCO4C1 |
| 297 | ADH1C |
| 298 | GNAS |
| 299 | LSM6 |
| 300 | SLCO1B3 |
| 301 | SERPINA6 |
| 302 | TPH1 |
| 303 | TPH2 |
| 304 | GSK3B |
| 305 | HBA1 |
| 306 | HBA2 |
| 307 | MAN2A1 |
| 308 | PMS2 |
| 309 | ABCA1 |
| 310 | NAGS |
| 311 | PRMT1 |
| 312 | PYGL |
| 313 | CYP3A4 |
| 314 | MMACHC |
| 315 | HNMT |
| 316 | TGM2 |
| 317 | PYGM |
| 318 | CYP3A5 |
| 319 | TGM3 |
| 320 | RNASE1 |
| 321 | RNASE2 |
| 322 | CDC42 |
| 323 | CYP2A13 |
| 324 | SLC10A1 |
| 325 | PRMT3 |
| 326 | RNASE3 |
| 327 | SLC10A2 |
| 328 | RNASE4 |
| 329 | SAR1B |
| 330 | SDHA |
| 331 | AKT1 |
| 332 | EGLN1 |
| 333 | SDHB |
| 334 | EGLN2 |
| 335 | SDHC |
| 336 | EGLN3 |
| 337 | SDHD |
| 338 | SLC10A6 |
| 339 | TXNRD1 |
| 340 | TGM1 |
| 341 | SMOX |
| 342 | TGM4 |
| 343 | CYP3A7 |
| 344 | MCCC1 |
| 345 | TGM5 |
| 346 | MCCC2 |
| 347 | TGM6 |
| 348 | CHEK1 |
| 349 | RPL13A |
| 350 | TGM7 |
| 351 | CYP11B1 |
| 352 | PIK3R1 |
| 353 | CYP11B2 |
| 354 | PIK3R2 |
| 355 | BLVRA |
| 356 | EEF2 |
| 357 | PIK3R3 |
| 358 | BLVRB |
| 359 | CYTH2 |
| 360 | CYTH3 |
| 361 | METAP2 |
| 362 | UPP1 |
| 363 | UPP2 |
| 364 | PSAP |
| 365 | TARS2 |
| 366 | MTOR |
| 367 | PHOSPHO1 |
| 368 | PAPSS1 |
| 369 | PRKAR1A |
| 370 | ENAH |
| 371 | ADRB1 |
| 372 | LDHA |
| 373 | KIF1A |
| 374 | ADRB2 |
| 375 | ADRB3 |
| 376 | LDHB |
| 377 | PDHB |
| 378 | LDHC |
| 379 | LARS2 |
| 380 | POU5F1 |
| 381 | ALDH1A1 |
| 382 | YWHAE |
| 383 | ALDH1A2 |
| 384 | ALDH1A3 |
| 385 | PDXK |
| 386 | HSPA2 |
| 387 | PITPNA |
| 388 | XDH |
| 389 | NCOA1 |
| 390 | HSPA5 |
| 391 | FTCD |
| 392 | NCOA2 |
| 393 | NFKB1 |
| 394 | NFKB2 |
| 395 | RAB9A |
| 396 | NCOA5 |
| 397 | RAB5A |
| 398 | HSPA8 |
| 399 | PSAT1 |
| 400 | ADCY1 |
| 401 | LPA |
| 402 | ADCY2 |
| 403 | CA1 |
| 404 | ADCY5 |
| 405 | RFK |
| 406 | PLA2G2A |
| 407 | CA2 |
| 408 | NFATC1 |
| 409 | LTF |
| 410 | CA3 |
| 411 | RHOA |
| 412 | AHCY |
| 413 | CYP4A11 |
| 414 | CA4 |
| 415 | HRH1 |
| 416 | PLA2G2D |
| 417 | HRH2 |
| 418 | DRD1 |
| 419 | CA6 |
| 420 | GABRA1 |
| 421 | HRH3 |
| 422 | DRD2 |
| 423 | CA7 |
| 424 | GABRA2 |
| 425 | HRH4 |
| 426 | PRKAG1 |
| 427 | DRD3 |
| 428 | GABRA3 |
| 429 | PRKAG2 |
| 430 | DRD4 |
| 431 | CA9 |
| 432 | ABAT |
| 433 | GABRA4 |
| 434 | CRABP1 |
| 435 | GABRA5 |
| 436 | CRABP2 |
| 437 | GABRA6 |
| 438 | SLC5A1 |
| 439 | SLC5A2 |
| 440 | NNMT |
| 441 | PTGR1 |
| 442 | COX7B |
| 443 | OPRK1 |
| 444 | COX7C |
| 445 | SLC5A6 |
| 446 | OAT |
| 447 | RPL26L1 |
| 448 | PDE7A |
| 449 | SLC5A7 |
| 450 | PDE7B |
| 451 | PDE3B |
| 452 | CAD |
| 453 | GLUD1 |
| 454 | QDPR |
| 455 | GLUD2 |
| 456 | PRKACA |
| 457 | UGT2B4 |
| 458 | PPP5C |
| 459 | CFTR |
| 460 | ART1 |
| 461 | UGT2B7 |
| 462 | KDM5D |
| 463 | ARL1 |
| 464 | RSL24D1 |
| 465 | ARL3 |
| 466 | ACSL1 |
| 467 | CAT |
| 468 | MB |
| 469 | ACSL3 |
| 470 | ACSL4 |
| 471 | APH1A |
| 472 | CYP1B1 |
| 473 | EIF4E |
| 474 | TREM1 |
| 475 | MGAT1 |
| 476 | CYP26C1 |
| 477 | ACOT13 |
| 478 | OPLAH |
| 479 | VASP |
| 480 | GNPDA1 |
| 481 | ACO2 |
| 482 | AR |
| 483 | B4GALT1 |
| 484 | GAPDH |
| 485 | BCKDK |
| 486 | GFPT2 |
| 487 | NOS1 |
| 488 | ATP5C1 |
| 489 | PASS2 |
| 490 | NOS3 |
| 491 | RXRA |
| 492 | CYTB |
| 493 | PNMT |
| 494 | CPA1 |
| 495 | PLOD1 |
| 496 | PLOD2 |
| 497 | RXRG |
| 498 | PLOD3 |
| 499 | PKLR |
| 500 | SIGLEC1 |
| 501 | SCN7A |
| 502 | DHRS3 |
| 503 | SCN3A |
| 504 | DHRS4 |
| 505 | SCN3B |
| 506 | SIGLEC7 |
| 507 | TERT |
| 508 | DAPP1 |
| 509 | UBA1 |
| 510 | RAB11A |
| 511 | PGD |
| 512 | GSPT1 |
| 513 | PLCD1 |
| 514 | ACADSB |
| 515 | SPR |
| 516 | NDST1 |
| 517 | GAD1 |
| 518 | GAD2 |
| 519 | APOA1 |
| 520 | SDS |
| 521 | POR |
| 522 | GRID1 |
| 523 | GRID2 |
| 524 | PVALB |
| 525 | SULT1E1 |
| 526 | PGR |
| 527 | SULT1A1 |
| 528 | JUN |
| 529 | HMOX1 |
| 530 | NFKBIA |
| 531 | HMOX2 |
| 532 | GPER |
| 533 | AMY2A |
| 534 | RARRES1 |
| 535 | GMPR |
| 536 | AMY2B |
| 537 | SLC25A20 |
| 538 | DHCR7 |
| 539 | GMPS |
| 540 | SLC25A22 |
| 541 | GMDS |
| 542 | ANG |
| 543 | DPYD |
| 544 | C14 |
| 545 | GALE |
| 546 | FOLH1 |
| 547 | DCK |
| 548 | GLS |
| 549 | GPT |
| 550 | MTHFD1 |
| 551 | SLC25A29 |
| 552 | MTHFD2 |
| 553 | ESR1 |
| 554 | ESR2 |
| 555 | GATM |
| 556 | ABO |
| 557 | CACNA1A |
| 558 | VKORC1 |
| 559 | CACNA1B |
| 560 | TLR3 |
| 561 | CACNA1C |
| 562 | CACNA1D |
| 563 | TLR4 |
| 564 | YARS2 |
| 565 | DECR1 |
| 566 | CYP2C18 |
| 567 | CACNA1F |
| 568 | ETFDH |
| 569 | CYP2C19 |
| 570 | TLR7 |
| 571 | CACNA1G |
| 572 | AMD1 |
| 573 | PIM1 |
| 574 | CACNA1H |
| 575 | CACNA1I |
| 576 | MMP7 |
| 577 | CALY |
| 578 | MMP9 |
| 579 | RPL23 |
| 580 | PPP2CA |
| 581 | CYP2D6 |
| 582 | CYP27A1 |
| 583 | PPP2CB |
| 584 | TUBD1 |
| 585 | IGF1 |
| 586 | PCK1 |
| 587 | ERAP1 |
| 588 | CACNA1S |
| 589 | ERAP2 |
| 590 | HIF1A |
| 591 | GANAB |
| 592 | TUBA4A |
| 593 | HADH |
| 594 | P4HA1 |
| 595 | PDSS1 |
| 596 | P4HA2 |
| 597 | PRODH |
| 598 | RAD51 |
| 599 | PRLR |
| 600 | SCN11A |
| 601 | SLC22A1 |
| 602 | SLC22A2 |
| 603 | AKR1C1 |
| 604 | ASNS |
| 605 | ENPEP |
| 606 | SLC22A3 |
| 607 | AKR1C2 |
| 608 | SLC22A4 |
| 609 | AKR1C3 |
| 610 | LDHAL6A |
| 611 | AKR1C4 |
| 612 | SLC22A5 |
| 613 | ALDH3A1 |
| 614 | LDHAL6B |
| 615 | SLC22A6 |
| 616 | FADS1 |
| 617 | ALDH3A2 |
| 618 | SLC22A7 |
| 619 | FADS2 |
| 620 | CNR1 |
| 621 | ND1 |
| 622 | SLC22A8 |
| 623 | CNR2 |
| 624 | ND2 |
| 625 | PCCA |
| 626 | BCKDHA |
| 627 | ND3 |
| 628 | PCCB |
| 629 | BCKDHB |
| 630 | ND4 |
| 631 | ND5 |
| 632 | FGF1 |
| 633 | TNF |
| 634 | GSTO1 |
| 635 | EDNRA |
| 636 | UCK2 |
| 637 | TNFSF11 |
| 638 | HNF1A |
| 639 | CES1 |
| 640 | CES2 |
| 641 | CACNG1 |
| 642 | CBR1 |
| 643 | CTPS |
| 644 | CTDSP1 |
| 645 | SLC7A1 |
| 646 | SLC7A2 |
| 647 | HBB |
| 648 | ITPKA |
| 649 | SLC7A3 |
| 650 | SF3B3 |
| 651 | SLC7A4 |
| 652 | SLC7A5 |
| 653 | FFAR1 |
| 654 | PFKFB1 |
| 655 | SLC7A7 |
| 656 | RALA |
| 657 | ASRGL1 |
| 658 | SLC7A8 |
| 659 | PFKFB4 |
| 660 | CHDH |
| 661 | AMHR2 |
| 662 | BPI |
| 663 | SLC19A1 |
| 664 | BTK |
| 665 | SLC19A2 |
| 666 | CHRND |
| 667 | HS3ST1 |
| 668 | CHRNA1 |
| 669 | SAT1 |
| 670 | UGT1A1 |
| 671 | CHRNE |
| 672 | CHRNA2 |
| 673 | SAT2 |
| 674 | PRSS1 |
| 675 | CHRNA3 |
| 676 | SLCO1C1 |
| 677 | UGT1A3 |
| 678 | HTR1A |
| 679 | PRSS2 |
| 680 | CHRNG |
| 681 | CHRNA4 |
| 682 | UGT1A4 |
| 683 | KAT5 |
| 684 | APAF1 |
| 685 | PRSS3 |
| 686 | CHRNA5 |
| 687 | STK17B |
| 688 | CHRNA6 |
| 689 | UGT1A6 |
| 690 | NDUFA10 |
| 691 | TPI1 |
| 692 | TRDMT1 |
| 693 | UGT1A7 |
| 694 | ATP6 |
| 695 | CHRNA7 |
| 696 | NDUFA11 |
| 697 | HTR1B |
| 698 | UGT1A8 |
| 699 | NDUFA12 |
| 700 | CHRNA9 |
| 701 | UGT1A9 |
| 702 | NDUFA13 |
| 703 | HTR1D |
| 704 | PET112 |
| 705 | ABCB1 |
| 706 | PMP2 |
| 707 | HLA-A |
| 708 | HLA-B |
| 709 | HLA-C |
| 710 | ABCB4 |
| 711 | TP53 |
| 712 | COX7A1 |
| 713 | PPCDC |
| 714 | ITPA |
| 715 | CYP17A1 |
| 716 | BCAT1 |
| 717 | BCAT2 |
| 718 | HSD3B1 |
| 719 | HSD3B2 |
| 720 | PPIA |
| 721 | PPIB |
| 722 | TNFRSF10B |
| 723 | PPIC |
| 724 | CPT1A |
| 725 | SLC29A1 |
| 726 | TARSL2 |
| 727 | SLC29A2 |
| 728 | PPIF |
| 729 | PPIG |
| 730 | PPIH |
| 731 | TUBB2B |
| 732 | MTTP |
| 733 | OSTalpha |
| 734 | HMGCL |
| 735 | NQO1 |
| 736 | BCL2 |
| 737 | NQO2 |
| 738 | ACCN3 |
| 739 | RPL10L |
| 740 | SLC16A10 |
| 741 | GRM1 |
| 742 | PRKAR2B |
| 743 | COX1 |
| 744 | GRM4 |
| 745 | GOT1 |
| 746 | HMGCR |
| 747 | COX2 |
| 748 | UGT1A10 |
| 749 | GOT2 |
| 750 | PSMA1 |
| 751 | KIF2C |
| 752 | PGRMC1 |
| 753 | PPAT |
| 754 | GRM7 |
| 755 | ATP1A1 |
| 756 | ALDH1B1 |
| 757 | GRM8 |
| 758 | ATP1A2 |
| 759 | ATP1A3 |
| 760 | FABP1 |
| 761 | PSMA4 |
| 762 | AZGP1 |
| 763 | PDE10A |
| 764 | GLS2 |
| 765 | GLO1 |
| 766 | FABP2 |
| 767 | PSMA5 |
| 768 | FABP3 |
| 769 | PIK3CA |
| 770 | FABP4 |
| 771 | FABP5 |
| 772 | IL2 |
| 773 | FABP6 |
| 774 | FABP7 |
| 775 | IL6 |
| 776 | EXTL2 |
| 777 | PIK3CG |
| 778 | UAP1 |
| 779 | PDHA1 |
| 780 | BCHE |
| 781 | PDHA2 |
| 782 | NPEPPS |
| 783 | CAMK2G |
| 784 | CCT3 |
| 785 | GCH1 |
| 786 | GABRR1 |
| 787 | PTPN2 |
| 788 | SLC44A1 |
| 789 | CUBN |
| 790 | GNRHR |
| 791 | SLC44A2 |
| 792 | SLC44A3 |
| 793 | GABRB1 |
| 794 | SLC44A4 |
| 795 | GABRB2 |
| 796 | GABRB3 |
| 797 | UMPS |
| 798 | COX8A |
| 799 | CRYZ |
| 800 | CRAT |
| 801 | PTGS1 |
| 802 | OPRL1 |
| 803 | PTGS2 |
| 804 | IL4I1 |
| 805 | SLC2A1 |
| 806 | CYP19A1 |
| 807 | HRSP12 |
| 808 | DLG4 |
| 809 | SLC2A2 |
| 810 | OPRD1 |
| 811 | SLC2A3 |
| 812 | PDE4A |
| 813 | GCLC |
| 814 | FECH |
| 815 | NDUFA1 |
| 816 | SLC2A4 |
| 817 | PDE4B |
| 818 | NDUFA2 |
| 819 | SLC2A5 |
| 820 | PDE4C |
| 821 | F2 |
| 822 | NDUFA3 |
| 823 | SLC2A6 |
| 824 | PDE4D |
| 825 | LYZ |
| 826 | NDUFA4 |
| 827 | SLC2A7 |
| 828 | NDUFA5 |
| 829 | SLC2A8 |
| 830 | NDUFA6 |
| 831 | SLC2A9 |
| 832 | ANXA1 |
| 833 | GCDH |
| 834 | NDUFA7 |
| 835 | SLCO2A1 |
| 836 | FOS |
| 837 | F7 |
| 838 | NDUFA8 |
| 839 | NDUFA9 |
| 840 | ACVR1 |
| 841 | SEC61G |
| 842 | PTGER2 |
| 843 | F9 |
| 844 | GCLM |
| 845 | ENOX2 |
| 846 | PTGER3 |
| 847 | PTGER4 |
| 848 | GFER |
| 849 | CTRB1 |
| 850 | CASP3 |
| 851 | AOX1 |
| 852 | RUVBL2 |
| 853 | NARS2 |
| 854 | EFTUD1 |
| 855 | TNFSF13B |
| 856 | TTPA |
| 857 | PKM2 |
| 858 | FKBP1A |
| 859 | HRAS |
| 860 | TRAPPC3 |
| 861 | DCTD |
| 862 | SRPK2 |
| 863 | HLCS |
| 864 | ACP1 |
| 865 | ARAF |
| 866 | PCYT1A |
| 867 | PKIA |
| 868 | PCYT1B |
| 869 | ATP8A1 |
| 870 | DCXR |
| 871 | RRM1 |
| 872 | RRM2 |
| 873 | DCPS |
| 874 | ARL5A |
| 875 | GSG2 |
| 876 | ALDH5A1 |
| 877 | HIBCH |
| 878 | KCNH2 |
| 879 | ARL5B |
| 880 | CYB5R1 |
| 881 | CYB5R3 |
| 882 | CPB1 |
| 883 | ACTB |
| 884 | MAT1A |
| 885 | SCN8A |
| 886 | SCN4A |
| 887 | ACHE |
| 888 | SCN4B |
| 889 | NDUFA4L2 |
| 890 | BBOX1 |
| 891 | GNAT1 |
| 892 | ACACA |
| 893 | SYK |
| 894 | ACACB |
| 895 | AFG3L2 |
| 896 | PECR |
| 897 | PTH |
| 898 | PLG |
| 899 | ORM1 |
| 900 | ORM2 |
| 901 | AK1 |
| 902 | ALDH2 |
| 903 | SMO |
| 904 | AK2 |
| 905 | ALAS2 |
| 906 | CCBL1 |
| 907 | SUCNR1 |
| 908 | CCBL2 |
| 909 | SOD1 |
| 910 | SMS |
| 911 | AK8 |
| 912 | KLK1 |
| 913 | AMPD1 |
| 914 | GAA |
| 915 | GRIA1 |
| 916 | KLK3 |
| 917 | GRIA2 |
| 918 | CA12 |
| 919 | SULT1B1 |
| 920 | GRIA3 |
| 921 | GRIA4 |
| 922 | GIF |
| 923 | CA14 |
| 924 | DLD |
| 925 | DDC |
| 926 | DHCR24 |
| 927 | GDNF |
| 928 | SLC25A32 |
| 929 | LEPRE1 |
| 930 | STMN4 |
| 931 | CMAS |
| 932 | UGT3A1 |
| 933 | ACE |
| 934 | EIF2S3 |
| 935 | CA5A |
| 936 | ASL |
| 937 | CA5B |
| 938 | ASS1 |
| 939 | DDO |
| 940 | TOP1 |
| 941 | EPN1 |
| 942 | PTK2B |
| 943 | GGCX |
| 944 | PLK1 |
| 945 | DGKA |
| 946 | CANT1 |
| 947 | GAMT |
| 948 | DGKD |
| 949 | MTFMT |
| 950 | TUBB |
| 951 | CYP2E1 |
| 952 | DGKG |
| 953 | KAT2A |
| 954 | IGHG1 |
| 955 | LCMT1 |
| 956 | KAT2B |
| 957 | IGHG2 |
| 958 | LCMT2 |
| 959 | CYP27B1 |
| 960 | CYP2A6 |
| 961 | TUBE1 |
| 962 | POLB |
| 963 | RPL37 |
| 964 | TUFM |
| 965 | CYP24A1 |
| 966 | NR3C1 |
| 967 | NR3C2 |
| 968 | TUBA1A |
| 969 | TUBA1B |
| 970 | TK1 |
| 971 | TUBA1C |
| 972 | TK2 |
| 973 | POLK |
| 974 | RAP2A |
| 975 | NR0B1 |
| 976 | MMAA |
| 977 | SLC36A1 |
| 978 | MMAB |
| 979 | CMPK1 |
| 980 | PREP |
| 981 | AKR1D1 |
| 982 | HSD17B1 |
| 983 | PDPK1 |
| 984 | HSD17B2 |
| 985 | LRAT |
| 986 | HSD17B3 |
| 987 | ALDH3B1 |
| 988 | HSD17B4 |
| 989 | ALDH3B2 |
| 990 | EPRS |
| 991 | ABCC10 |
| 992 | NME1 |
| 993 | APRT |
| 994 | ABCC11 |
| 995 | NME2 |
| 996 | HSD17B6 |
| 997 | HSD17B7 |
| 998 | HCAR2 |
| 999 | HSD17B8 |
| 1000 | HCAR3 |
| 1001 | GABRD |
| 1002 | GABRE |
| 1003 | B3GAT1 |
| 1004 | CEBPB |
| 1005 | B3GAT3 |
| 1006 | LCTL |
| 1007 | HK1 |
| 1008 | GSTT1 |
| 1009 | GSTP1 |
| 1010 | PCTP |
| 1011 | MTNR1A |
| 1012 | MTNR1B |
| 1013 | PFAS |
| 1014 | GABRP |
| 1015 | GABRQ |
| 1016 | BHMT |
| 1017 | SLC46A1 |
| 1018 | NAE1 |
| 1019 | IDH3A |
| 1020 | ADORA1 |
| 1021 | IDH3B |
| 1022 | SUCLG1 |
| 1023 | SUCLG2 |
| 1024 | IDH3G |
| 1025 | SARS2 |
| 1026 | ITGA5 |
| 1027 | THADA |
| 1028 | SGK1 |
| 1029 | CHIT1 |
| 1030 | PHGDH |
| 1031 | HCK |
| 1032 | EVL |
| 1033 | PLA2G6 |
| 1034 | LEPREL1 |
| 1035 | LEPREL2 |
| 1036 | KCNMA1 |
| 1037 | SLCO4A1 |
| 1038 | HS3ST3A1 |
| 1039 | CHRNB1 |
| 1040 | HGS |
| 1041 | CHRNB2 |
| 1042 | LY96 |
| 1043 | PPP3R1 |
| 1044 | FAAH |
| 1045 | CHRNB3 |
| 1046 | NDUFAB1 |
| 1047 | CHRNB4 |
| 1048 | RDH11 |
| 1049 | SLC16A1 |
| 1050 | SLC16A2 |
| 1051 | RDH12 |
| 1052 | RDH13 |
| 1053 | SLC16A3 |
| 1054 | PNPLA8 |
| 1055 | PSG5 |
| 1056 | SLC16A4 |
| 1057 | HTR2A |
| 1058 | UQCR10 |
| 1059 | RDH14 |
| 1060 | CSNK2B |
| 1061 | CHAT |
| 1062 | UQCR11 |
| 1063 | TRPA1 |
| 1064 | SLC16A5 |
| 1065 | HTR2B |
| 1066 | SLC16A6 |
| 1067 | HTR2C |
| 1068 | SPTBN1 |
| 1069 | ABCG1 |
| 1070 | SLC16A7 |
| 1071 | TRPM7 |
| 1072 | ABCG2 |
| 1073 | ABCC1 |
| 1074 | TRPM8 |
| 1075 | ABCC2 |
| 1076 | ABCC3 |
| 1077 | ABCG5 |
| 1078 | ABCC4 |
| 1079 | HEXB |
| 1080 | GALK1 |
| 1081 | ABCC5 |
| 1082 | ITGAL |
| 1083 | ABCC6 |
| 1084 | ABCG8 |
| 1085 | COX4I1 |
| 1086 | ABCC8 |
| 1087 | ABCC9 |
| 1088 | PGK1 |
| 1089 | TYMP |
| 1090 | ATIC |
| 1091 | OBP2A |
| 1092 | DNMT1 |
| 1093 | TYMS |
| 1094 | CTBP1 |
| 1095 | MED1 |
| 1096 | CDIPT |
| 1097 | MAPK1 |
| 1098 | GUK1 |
| 1099 | NR1H4 |
| 1100 | MAPK3 |
| 1101 | ALDH7A1 |
| 1102 | LGALS1 |
| 1103 | LGALS2 |
| 1104 | ADORA2A |
| 1105 | MTAP |
| 1106 | LGALS3 |
| 1107 | ADORA2B |
| 1108 | PARS2 |
| 1109 | LGALS7 |
| 1110 | COQ2 |
| 1111 | PSMB1 |
| 1112 | ASNA1 |
| 1113 | PSMB2 |
| 1114 | TRIM13 |
| 1115 | INSR |
| 1116 | LNPEP |
| 1117 | COQ6 |
| 1118 | LGSN |
| 1119 | PSMB7 |
| 1120 | PGCP |
| 1121 | RAB7A |
| 1122 | DNPEP |
| 1123 | NT5E |
| 1124 | NT5C2 |
| 1125 | PLA2G4A |
| 1126 | PAEP |
| 1127 | NUDT9 |
| 1128 | GABRG1 |
| 1129 | RHO |
| 1130 | QPRT |
| 1131 | GABRG2 |
| 1132 | GABRG3 |
| 1133 | RHEB |
| 1134 | CRBN |
| 1135 | PRKAA1 |
| 1136 | UGCG |
| 1137 | PRKAA2 |
| 1138 | NDUFV1 |
| 1139 | COX5A |
| 1140 | RDX |
| 1141 | NDUFV2 |
| 1142 | EPHA2 |
| 1143 | OPRM1 |
| 1144 | GLTP |
| 1145 | COX5B |
| 1146 | NDUFV3 |
| 1147 | HINT1 |
| 1148 | SIGMAR1 |
| 1149 | COMT |
| 1150 | PDE5A |
| 1151 | NDUFB1 |
| 1152 | NDUFB2 |
| 1153 | NDUFB3 |
| 1154 | SQLE |
| 1155 | SMPD3 |
| 1156 | NDUFB4 |
| 1157 | SMPD4 |
| 1158 | NDUFB5 |
| 1159 | ACYP2 |
| 1160 | NDUFB6 |
| 1161 | SNCA |
| 1162 | FHIT |
| 1163 | NDUFB7 |
| 1164 | SLCO2B1 |
| 1165 | NDUFB8 |
| 1166 | CKM |
| 1167 | GLRA1 |
| 1168 | NDUFB9 |
| 1169 | GLRA2 |
| 1170 | GLRA3 |
| 1171 | ELOVL3 |
| 1172 | BST1 |
| 1173 | RETSAT |
| 1174 | ARF1 |
| 1175 | ELOVL4 |
| 1176 | TNK2 |
| 1177 | GUCA1A |
| 1178 | CEPT1 |
| 1179 | ARF4 |
| 1180 | OXCT1 |
| 1181 | ARF6 |
| 1182 | OXCT2 |
| 1183 | GC |
| 1184 | MYH14 |
| 1185 | SELE |
| 1186 | PPARA |
| 1187 | SI |
| 1188 | AADAT |
| 1189 | PPARD |
| 1190 | SCARB1 |
| 1191 | PPARG |
| 1192 | RPL23A |
| 1193 | CYP26A1 |
| 1194 | MAPK10 |
| 1195 | PPP1CC |
| 1196 | SELP |
| 1197 | MAPK12 |
| 1198 | NR2F1 |
| 1199 | PTEN |
| 1200 | CS |
| 1201 | GRIN2A |
| 1202 | ECE1 |
| 1203 | GRIN2B |
| 1204 | ECI2 |
| 1205 | SLC38A3 |
| 1206 | GRIN2C |
| 1207 | SCPEP1 |
| 1208 | GRIN2D |
| 1209 | LTA4H |
| 1210 | PRKCA |
| 1211 | PTAFR |
| 1212 | PRKCB |
| 1213 | ACAT2 |
| 1214 | ATP5A1 |
| 1215 | HPGDS |
| 1216 | PRKCD |
| 1217 | PHYH |
| 1218 | CPS1 |
| 1219 | PRKCG |
| 1220 | ESRRA |
| 1221 | SREBF1 |
| 1222 | SREBF2 |
| 1223 | CYBA |
| 1224 | ESRRB |
| 1225 | CYBB |
| 1226 | IFNG |
| 1227 | ACAD8 |
| 1228 | MAT2A |
| 1229 | ATP6V1C1 |
| 1230 | INPP5B |
| 1231 | SCN9A |
| 1232 | SRC |
| 1233 | SCN5A |
| 1234 | ESRRG |
| 1235 | SCN1A |
| 1236 | SCN1B |
| 1237 | NSDHL |
| 1238 | CDO1 |
| 1239 | GNAI1 |
| 1240 | CDK2 |
| 1241 | IMPA1 |
| 1242 | VCP |
| 1243 | PAH, |
| 1244 | HSD17B10 |
| 1245 | HIF1AN |
| 1246 | SRR |
| 1247 | HSD17B11 |
| 1248 | CDK6 |
| 1249 | ACADM |
| 1250 | P2RY11 |
| 1251 | GM2A |
| 1252 | GRIN1 |
| 1253 | VKORC1L1 |
| 1254 | PAM |
| 1255 | IFNB1 |
| 1256 | P2RY12 |
| 1257 | MC1R |
| 1258 | GBA |
| 1259 | AIMP1 |
| 1260 | MPO |
| 1261 | SLC6A2 |
| 1262 | SLC6A3 |
| 1263 | ACADS |
| 1264 | SLC6A4 |
| 1265 | MTR |
| 1266 | SLC6A5 |
| 1267 | SLC6A7 |
| 1268 | ACVRL1 |
| 1269 | ALB |
| 1270 | ADA |
| 1271 | DHODH |
| 1272 | TPSAB1 |
| 1273 | OXTR |
| 1274 | GANC |
| 1275 | FOLR1 |
| 1276 | FOLR2 |
| 1277 | SLC18A1 |
| 1278 | FOLR3 |
| 1279 | SLC18A2 |
| 1280 | HPGD |
| 1281 | ALK |
| 1282 | HADHA |
| 1283 | NCAN |
| 1284 | DUT |
| 1285 | ADK |
| 1286 | DAO |
| 1287 | APP |
| 1288 | MAN1B1 |
| 1289 | AHR |
| 1290 | GARS |
| 1291 | GART |
| 1292 | CSNK1G2 |
| 1293 | CYP2R1 |
| 1294 | PLD1 |
| 1295 | PLD2 |
| 1296 | NADSYN1 |
| 1297 | COX6A2 |
| 1298 | CYP2J2 |
| 1299 | PLD6 |
| 1300 | CYP2B6 |
| 1301 | MAPKAPK2 |
| 1302 | ADRA1A |
| 1303 | POMC |
| 1304 | TUBB1 |
| 1305 | MAPKAPK3 |
| 1306 | ADRA1B |
| 1307 | SLC28A1 |
| 1308 | ASPH |
| 1309 | IDO1 |
| 1310 | MARS2 |
| 1311 | ADRA1D |
| 1312 | APOD |
| 1313 | APOE |
| 1314 | SLC28A3 |
| 1315 | WNT5A |
| 1316 | ALDH9A1 |
| 1317 | HIBADH |
| 1318 | PISD |
| 1319 | EARS2 |
| 1320 | APOM |
| 1321 | VEGFA |
| 1322 | AKR1A1 |
| 1323 | VANGL2 |
| 1324 | POLE2 |
| 1325 | POLA1 |
| 1326 | APCS |
| 1327 | POLE3 |
| 1328 | POLE4 |
| 1329 | ISG20 |
| 1330 | SYN1 |
| 1331 | IGFBP3 |
| 1332 | F13A1 |
| 1333 | CXCR4 |
| 1334 | AGXT |
| 1335 | TTL |
| 1336 | GSTM3 |
| 1337 | C22orf28 |
| 1338 | GSTA2 |
| 1339 | NMNAT1 |
| 1340 | IMPDH1 |
| 1341 | TTR |
| 1342 | LCAT |
| 1343 | NMNAT3 |
| 1344 | IMPDH2 |
| 1345 | HTR6 |
| 1346 | SLC43A1 |
| 1347 | MARS |
| 1348 | HTR7 |
| 1349 | P4HB |
| 1350 | GNRHR2 |
| 1351 | FPGS |
| 1352 | SULT2A1 |
| 1353 | ITGB2 |
| 1354 | ITGB3 |
| 1355 | RARA |
| 1356 | RARB |
| 1357 | NNT |
| 1358 | SIRT3 |
| 1359 | HDAC2 |
| 1360 | EGF |
| 1361 | SIRT5 |
| 1362 | SLC1A1 |
| 1363 | SLC1A2 |
| 1364 | SLC1A3 |
| 1365 | RARG |
| 1366 | SLC1A5 |
| 1367 | HDAC9 |
| 1368 | OAZ1 |
| 1369 | SLC1A6 |
| 1370 | KYNU |
| 1371 | OAZ2 |
| 1372 | SLC1A7 |
| 1373 | ODC1 |
| 1374 | PSMB10 |
| 1375 | ADRBK1 |
| 1376 | OAZ3 |
| 1377 | ADRBK2 |
| 1378 | NOS2A |
| 1379 | ATP5B |
| 1380 | TRPV1 |
| 1381 | TRPV3 |
| 1382 | KIAA1310 |
| 1383 | SLCO1A2 |
| 1384 | ATF1 |
| 1385 | KCNJ10 |
| 1386 | GAPDHS |
| 1387 | ATF2 |
| 1388 | KCNJ11 |
| 1389 | TPK1 |
| 1390 | HTR3A |
| 1391 | ATF3 |
| 1392 | KCNJ12 |
| 1393 | PSENEN |
| 1394 | ATF4 |
| 1395 | PTDSS1 |
| 1396 | NARS |
| 1397 | SLC13A1 |
| 1398 | ATF5 |
| 1399 | KCNJ14 |
| 1400 | PTDSS2 |
| 1401 | SLC13A2 |
| 1402 | ATF6 |
| 1403 | KCNJ15 |
| 1404 | SLC13A3 |
| 1405 | ATF7 |
| 1406 | OSTBETA |
| 1407 | ACOX1 |
| 1408 | CSNK2A1 |
| 1409 | G6PD |
| 1410 | CACNA2D1 |
| 1411 | SMARCA5 |
| 1412 | SEC14L2 |
| 1413 | OGDH |
| 1414 | DNTT |
| 1415 | CACNA2D2 |
| 1416 | SEC14L4 |
| 1417 | ERO1LB |
| 1418 | CACNA2D3 |
| 1419 | PPP3CA |
| 1420 | PSPH |
| 1421 | SDSL |
| 1422 | S100B |
| 1423 | PYCR1 |
| 1424 | TDO2 |
| 1425 | PYCR2 |
| 1426 | PDK4 |
| 1427 | CYP11A1 |
| 1428 | HOXA10 |
| 1429 | PRDX5 |
| 1430 | EEA1 |
| 1431 | AKR1B10 |
| 1432 | DHFR |
| 1433 | NR1I2 |
| 1434 | ABL1 |
| 1435 | NR1I3 |
| 1436 | SNRPA |
| 1437 | ABL2 |
| 1438 | MBL2 |
| 1439 | SARS |
| 1440 | KARS |
| 1441 | SLC23A1 |
| 1442 | MTRR |
| 1443 | S100P |
| 1444 | CYP3A43 |
| 1445 | SRD5A1 |
| 1446 | ALDH4A1 |
| 1447 | PARP1 |
| 1448 | SRD5A2 |
| 1449 | SRD5A3 |
| 1450 | H6PD |
| 1451 | ALDH1L1 |
| 1452 | RND3 |
| 1453 | ALOX5 |
| 1454 | GPRC5A |
