# Supplementary material for: Synergistic Effects of Erzhi Pill Combined With Methotrexate on Osteoblasts Mediated via the Wnt1/LRP5/β-Catenin Signaling Pathway in Collagen-Induced Arthritis Rats
Source: Front Pharmacol. 2020 Mar 11;11:228. doi: 10.3389/fphar.2020.00228 (PMC7079734; doi:10.3389/fphar.2020.00228)
Supplement: Supplementary file 6 [file Table_5.docx]

Supplementary Material

# Supplementary Table 5. The targets of methotrexate (MTX)

| Number | Targets name |
| --- | --- |
| 1 | 5-hydroxytryptamine receptor 2C |
| 2 | Amyloid-beta precursor protein |
| 3 | Acetylcholinesterase |
| 4 | Serum albumin |
| 5 | Amine oxidase [flavin-containing] B |
| 6 | Carbonic anhydrase 2 |
| 7 | Cannabinoid receptor 1 |
| 8 | 72 kDa type IV collagenase |
| 9 | Matrilysin |
| 10 | Cytochrome P450 2E1 |
| 11 | D(2) dopamine receptor |
| 12 | D(1A) dopamine receptor |
| 13 | Dihydrofolate reductase |
| 14 | Receptor tyrosine-protein kinase erbB-2 |
| 15 | Endothelin-1 receptor |
| 16 | Glucocorticoid receptor |
| 17 | Histamine H2 receptor |
| 18 | 3-hydroxy-3-methylglutaryl-coenzyme A reductase |
| 19 | Tyrosine-protein kinase Fyn |
| 20 | Tyrosine-protein kinase Lck |
| 21 | Substance-P receptor |
| 22 | Sodium-dependent noradrenaline transporter |
| 23 | Trifunctional purine biosynthetic protein adenosine-3 |
| 24 | Thymidylate synthase |
| 25 | Adenosine receptor A1 |
| 26 | Sodium-dependent dopamine transporter |
| 27 | Sodium-dependent serotonin transporter |
| 28 | Cholecystokinin receptor type A |
| 29 | Parathyroid hormone/parathyroid hormone-related peptide receptor |
| 30 | Vasoactive intestinal polypeptide receptor 1 |
| 31 | Beta-3 adrenergic receptor |
| 32 | Thyrotropin-releasing hormone receptor |
| 33 | 5-hydroxytryptamine receptor 2A |
| 34 | Adenosine receptor A2a |
| 35 | Estrogen receptor |
| 36 | Folate receptor alpha |
| 37 | Histamine H1 receptor |
| 38 | Vasopressin V1a receptor |
| 39 | 5-hydroxytryptamine receptor 2B |
| 40 | Alpha-1D adrenergic receptor |
| 41 | C-C chemokine receptor type 2 |
| 42 | D(3) dopamine receptor |
| 43 | Solute carrier organic anion transporter family member 1A2 |
| 44 | Alpha-2A adrenergic receptor |
| 45 | C-X-C chemokine receptor type 2 |
| 46 | 5-hydroxytryptamine receptor 6 |
| 47 | Type-2 angiotensin II receptor |
| 48 | C-C chemokine receptor type 4 |
| 49 | C-C chemokine receptor type 5 |
| 50 | Folylpolyglutamate synthase, mitochondrial |
| 51 | B2 bradykinin receptor |
| 52 | Prostaglandin G/H synthase 2 |
| 53 | Estrogen receptor beta |
| 54 | Cytochrome P450 2C9 |
| 55 | Reduced folate transporter |
| 56 | Solute carrier organic anion transporter family member 1B1 |
| 57 | Glutathione reductase, mitochondrial |
| 58 | caspase-3 |
| 59 | Alpha-2C adrenergic receptor |
| 60 | Bifunctional purine biosynthesis protein PURH |
| 61 | Solute carrier organic anion transporter family member 1B3 |
| 62 | Solute carrier organic anion transporter family member 1C1 |
| 63 | Beta-1 adrenergic receptor |
| 64 | Cytochrome P450 2C19 |
| 65 | Broad substrate specificity ATP-binding cassette transporter ABCG2 |
| 66 | Solute carrier family 22 member 8 |
| 67 | Proton-coupled folate transporter |
| 68 | Solute carrier family 22 member 11 |
| 69 | Solute carrier organic anion transporter family member 4C1 |
| 70 | Sigma non-opioid intracellular receptor 1 |
| 71 | Phosphatidylinositol 4-kinase type 2-alpha |
| 72 | Solute carrier family 22 member 7 |
| 73 | Multidrug resistance-associated protein 7 |
| 74 | ATP-binding cassette sub-family C member 11 |
| 75 | Solute carrier family 22 member 6 |
| 76 | Cytochrome P450 2D6 |
| 77 | C-X-C chemokine receptor type 1 |
| 78 | cytochrome P450 family 3 subfamily A polypeptide 4 |
| 79 | Solute carrier family 2, facilitated glucose transporter member 1 |
| 80 | Cytochrome P450 3A4 |
| 81 | Folate receptor beta |
| 82 | Vascular endothelial growth factor receptor 1 |
| 83 | Multidrug resistance-associated protein 4 |
| 84 | Substance-K receptor |
| 85 | ATP-dependent translocase ABCB1 |
| 86 | Thromboxane-A synthase |
| 87 | Bile salt export pump |
| 88 | Matrix metalloproteinase-9 |
| 89 | parathyroid hormone/parathyroid hormone-related peptide receptor precursor |
| 90 | Protein-arginine deiminase type-4 |
| 91 | Multidrug resistance-associated protein 1 |
| 92 | Beta-2 adrenergic receptor |
| 93 | Solute carrier organic anion transporter family member 3A1 |
| 94 | Canalicular multispecific organic anion transporter 1 |
| 95 | Glutathione peroxidase 1 |
| 96 | Solute carrier organic anion transporter family member 2B1 |
| 97 | Solute carrier family 22 member 1 |
| 98 | Prostaglandin G/H synthase 1 |
| 99 | Cytosolic phospholipase A2 |
| 100 | Matrix metalloproteinase-14 |
| 101 | pregnane X nuclear receptor |
| 102 | Adenosine receptor A3 |
