# Supplementary material for: Synergistic Effects of Erzhi Pill Combined With Methotrexate on Osteoblasts Mediated via the Wnt1/LRP5/β-Catenin Signaling Pathway in Collagen-Induced Arthritis Rats
Source: Front Pharmacol. 2020 Mar 11;11:228. doi: 10.3389/fphar.2020.00228 (PMC7079734; doi:10.3389/fphar.2020.00228)
Supplement: Supplementary file 7 [file Table_6.docx]

Supplementary Material

# Supplementary Table 6. The rheumatoid arthritis (RA) genes

| Number | Gene name |
| --- | --- |
| 1 | A1BG |
| 2 | A2M |
| 3 | ABCB1 |
| 4 | ABCC1 |
| 5 | ABCC11 |
| 6 | ABCG2 |
| 7 | ABHD6 |
| 8 | ACAN |
| 9 | ACD |
| 10 | ACE |
| 11 | ACKR3 |
| 12 | ACOXL |
| 13 | ACP1 |
| 14 | ACP5 |
| 15 | ACTA2 |
| 16 | ADA |
| 17 | ADAD1 |
| 18 | ADAM10 |
| 19 | ADAM15 |
| 20 | ADAM17 |
| 21 | ADAMTS4 |
| 22 | ADAMTS5 |
| 23 | ADAMTS9 |
| 24 | ADAMTSL2 |
| 25 | ADGRG1 |
| 26 | ADIPOQ |
| 27 | ADIPOR1 |
| 28 | ADM |
| 29 | ADORA2A |
| 30 | ADRB2 |
| 31 | ADRB3 |
| 32 | AFF1 |
| 33 | AFF3 |
| 34 | AGER |
| 35 | AGXT2 |
| 36 | AHNAK2 |
| 37 | AHR |
| 38 | AHRR |
| 39 | AHSG |
| 40 | AICDA |
| 41 | AIF1 |
| 42 | AIRE |
| 43 | AKT1 |
| 44 | AKT2 |
| 45 | ALB |
| 46 | ALOX15 |
| 47 | ALOX5 |
| 48 | ALPL |
| 49 | AMH |
| 50 | AMPD1 |
| 51 | ANGPT1 |
| 52 | ANGPT2 |
| 53 | ANGPTL4 |
| 54 | ANKRD55 |
| 55 | ANO8 |
| 56 | ANPEP |
| 57 | ANXA1 |
| 58 | ANXA2 |
| 59 | ANXA3 |
| 60 | APLN |
| 61 | APOA1 |
| 62 | APOB |
| 63 | APOE |
| 64 | APOM |
| 65 | AR |
| 66 | ARAP1 |
| 67 | ARAP2 |
| 68 | AREG |
| 69 | ARG1 |
| 70 | ARHGEF3 |
| 71 | ARID5B |
| 72 | ARL14 |
| 73 | ARL15 |
| 74 | ARRB2 |
| 75 | ASAH1 |
| 76 | ASCC1 |
| 77 | ATG16L1 |
| 78 | ATG5 |
| 79 | ATIC |
| 80 | ATM |
| 81 | ATP1A1 |
| 82 | ATP4A |
| 83 | ATXN2 |
| 84 | AXL |
| 85 | B3GNT2 |
| 86 | B4GALT1 |
| 87 | BACH2 |
| 88 | BAD |
| 89 | BAG6 |
| 90 | BAK1 |
| 91 | BANK1 |
| 92 | BATF |
| 93 | BBC3 |
| 94 | BCHE |
| 95 | BCL2 |
| 96 | BCL2L1 |
| 97 | BCL2L11 |
| 98 | BCL6 |
| 99 | BGLAP |
| 100 | BHLHE41 |
| 101 | BID |
| 102 | BIRC3 |
| 103 | BIRC5 |
| 104 | BLK |
| 105 | BMP2 |
| 106 | BMP4 |
| 107 | BMP5 |
| 108 | BMX |
| 109 | BPI |
| 110 | BRAF |
| 111 | BRD1 |
| 112 | BSG |
| 113 | BST1 |
| 114 | BST2 |
| 115 | BTK |
| 116 | BTLA |
| 117 | BTN3A2 |
| 118 | BTNL2 |
| 119 | C1QA |
| 120 | C1QB |
| 121 | C1QBP |
| 122 | C1QC |
| 123 | C3 |
| 124 | C4B |
| 125 | C5 |
| 126 | C5AR1 |
| 127 | CABIN1 |
| 128 | CALCA |
| 129 | CALCRL |
| 130 | CALR |
| 131 | CARD8 |
| 132 | CASC3 |
| 133 | CASP12 |
| 134 | CASP3 |
| 135 | CASP5 |
| 136 | CASP8 |
| 137 | CASR |
| 138 | CASTOR1 |
| 139 | CAT |
| 140 | CAV1 |
| 141 | CBLN2 |
| 142 | CCL11 |
| 143 | CCL13 |
| 144 | CCL17 |
| 145 | CCL18 |
| 146 | CCL19 |
| 147 | CCL2 |
| 148 | CCL20 |
| 149 | CCL21 |
| 150 | CCL22 |
| 151 | CCL23 |
| 152 | CCL24 |
| 153 | CCL25 |
| 154 | CCL3 |
| 155 | CCL3L1 |
| 156 | CCL4 |
| 157 | CCL5 |
| 158 | CCL7 |
| 159 | CCN1 |
| 160 | CCN2 |
| 161 | CCN6 |
| 162 | CCR1 |
| 163 | CCR2 |
| 164 | CCR3 |
| 165 | CCR4 |
| 166 | CCR5 |
| 167 | CCR6 |
| 168 | CCR7 |
| 169 | CCR9 |
| 170 | CCRL2 |
| 171 | CD101 |
| 172 | CD14 |
| 173 | CD163 |
| 174 | CD1D |
| 175 | CD2 |
| 176 | CD200 |
| 177 | CD200R1 |
| 178 | CD209 |
| 179 | CD226 |
| 180 | CD244 |
| 181 | CD247 |
| 182 | CD27 |
| 183 | CD274 |
| 184 | CD276 |
| 185 | CD28 |
| 186 | CD34 |
| 187 | CD36 |
| 188 | CD38 |
| 189 | CD4 |
| 190 | CD40 |
| 191 | CD40LG |
| 192 | CD44 |
| 193 | CD5 |
| 194 | CD58 |
| 195 | CD6 |
| 196 | CD63 |
| 197 | CD68 |
| 198 | CD69 |
| 199 | CD80 |
| 200 | CD83 |
| 201 | CD84 |
| 202 | CD86 |
| 203 | CDH11 |
| 204 | CDH5 |
| 205 | CDK2 |
| 206 | CDK4 |
| 207 | CDK5RAP2 |
| 208 | CDK6 |
| 209 | CDKN1A |
| 210 | CDR1-AS |
| 211 | CENPJ |
| 212 | CEP57 |
| 213 | CERS6 |
| 214 | CETP |
| 215 | CFH |
| 216 | CFLAR |
| 217 | CFTR |
| 218 | CHAT |
| 219 | CHGA |
| 220 | CHI3L1 |
| 221 | CHID1 |
| 222 | CHRM3 |
| 223 | CHRNA7 |
| 224 | CHUK |
| 225 | CIITA |
| 226 | CIP2A |
| 227 | CIRBP |
| 228 | CKLF |
| 229 | CLDN5 |
| 230 | CLEC12A |
| 231 | CLEC16A |
| 232 | CLEC4A |
| 233 | CLEC4E |
| 234 | CLEC5A |
| 235 | CLEC7A |
| 236 | CLNK |
| 237 | CLOCK |
| 238 | CLU |
| 239 | CLYBL |
| 240 | CMKLR1 |
| 241 | CNR1 |
| 242 | CNR2 |
| 243 | CNTNAP4 |
| 244 | COG6 |
| 245 | COL1A1 |
| 246 | COL2A1 |
| 247 | COMP |
| 248 | COTL1 |
| 249 | COX2 |
| 250 | CP |
| 251 | CPA6 |
| 252 | CR1 |
| 253 | CREB1 |
| 254 | CRH |
| 255 | CRP |
| 256 | CRYGD |
| 257 | CSF1 |
| 258 | CSF1R |
| 259 | CSF2 |
| 260 | CSK |
| 261 | CST3 |
| 262 | CST5 |
| 263 | CTHRC1 |
| 264 | CTLA4 |
| 265 | CTNNB1 |
| 266 | CTSB |
| 267 | CTSK |
| 268 | CTSS |
| 269 | CUL1 |
| 270 | CX3CL1 |
| 271 | CX3CR1 |
| 272 | CXCL10 |
| 273 | CXCL12 |
| 274 | CXCL13 |
| 275 | CXCL16 |
| 276 | CXCL5 |
| 277 | CXCL8 |
| 278 | CXCL9 |
| 279 | CXCR2 |
| 280 | CXCR3 |
| 281 | CXCR4 |
| 282 | CXCR5 |
| 283 | CXCR6 |
| 284 | CYLD |
| 285 | CYP1A2 |
| 286 | CYP2C19 |
| 287 | CYP3A5 |
| 288 | CYP4F23P |
| 289 | CYP7B1 |
| 290 | DCK |
| 291 | DDA1 |
| 292 | DDAH1 |
| 293 | DDAH2 |
| 294 | DDIT3 |
| 295 | DDX39B |
| 296 | DDX6 |
| 297 | DEFA1 |
| 298 | DEFA3 |
| 299 | DEK |
| 300 | DHCR7 |
| 301 | DHFR |
| 302 | DHODH |
| 303 | DHRS3 |
| 304 | DKK1 |
| 305 | DLL1 |
| 306 | DNASE1 |
| 307 | DNASE1L3 |
| 308 | DNASE2 |
| 309 | DNMT3B |
| 310 | DOT1L |
| 311 | DPP4 |
| 312 | DRD3 |
| 313 | DVL2 |
| 314 | DYRK1A |
| 315 | E2F2 |
| 316 | EBAG9 |
| 317 | EBI3 |
| 318 | EDN1 |
| 319 | EFEMP1 |
| 320 | EFEMP2 |
| 321 | EFNB1 |
| 322 | EGF |
| 323 | EGFR |
| 324 | EGR1 |
| 325 | EIF4EBP1 |
| 326 | ELAVL1 |
| 327 | ELMO1 |
| 328 | EMCN |
| 329 | ENG |
| 330 | ENGASE |
| 331 | ENO1 |
| 332 | ENOX1 |
| 333 | ENPP2 |
| 334 | ENTPD1 |
| 335 | EOMES |
| 336 | EPAS1 |
| 337 | ERAP2 |
| 338 | ERBB2 |
| 339 | ERBB3 |
| 340 | ESR1 |
| 341 | ESR2 |
| 342 | ETFA |
| 343 | ETS1 |
| 344 | EZR |
| 345 | F11R |
| 346 | F2 |
| 347 | F2R |
| 348 | F2RL1 |
| 349 | F3 |
| 350 | FABP4 |
| 351 | FADD |
| 352 | FADS1 |
| 353 | FADS2 |
| 354 | FADS3 |
| 355 | FAM107A |
| 356 | FAM124A |
| 357 | FAP |
| 358 | FAS |
| 359 | FASLG |
| 360 | FCAR |
| 361 | FCGR1A |
| 362 | FCGR2A |
| 363 | FCGR2B |
| 364 | FCGR3A |
| 365 | FCGR3B |
| 366 | FCGRT |
| 367 | FCN1 |
| 368 | FCN2 |
| 369 | FCN3 |
| 370 | FCRL2 |
| 371 | FCRL3 |
| 372 | FCRL4 |
| 373 | FCRLA |
| 374 | FKBP5 |
| 375 | FLACC1 |
| 376 | FLG |
| 377 | FLI1 |
| 378 | FLT1 |
| 379 | FLT3 |
| 380 | FLT3LG |
| 381 | FN1 |
| 382 | FNDC5 |
| 383 | FOLR2 |
| 384 | FOS |
| 385 | FOXJ3 |
| 386 | FOXO3 |
| 387 | FOXP3 |
| 388 | FPGS |
| 389 | FPR1 |
| 390 | FPR2 |
| 391 | FSTL1 |
| 392 | FZD5 |
| 393 | GABARAPL3 |
| 394 | GADD45A |
| 395 | GALNT18 |
| 396 | GAS5 |
| 397 | GAS6 |
| 398 | GATA3 |
| 399 | GC |
| 400 | GCH1 |
| 401 | GDF15 |
| 402 | GDF5 |
| 403 | GFAP |
| 404 | GGH |
| 405 | GGT6 |
| 406 | GHRL |
| 407 | GHSR |
| 408 | GLCCI1 |
| 409 | GMCL2 |
| 410 | GNAQ |
| 411 | GNB3 |
| 412 | GP6 |
| 413 | GPI |
| 414 | GPSM3 |
| 415 | GPX1 |
| 416 | GRHL2 |
| 417 | GRM5 |
| 418 | GRN |
| 419 | GSDMB |
| 420 | GSK3B |
| 421 | GSN |
| 422 | GSTM1 |
| 423 | GSTP1 |
| 424 | GSTT1 |
| 425 | GTF2I |
| 426 | GUCY1B2 |
| 427 | GUSB |
| 428 | GZMB |
| 429 | H19 |
| 430 | HAMP |
| 431 | HAS1 |
| 432 | HAS2 |
| 433 | HAVCR1 |
| 434 | HAVCR2 |
| 435 | HDAC1 |
| 436 | HDAC2 |
| 437 | HDAC3 |
| 438 | HEXA |
| 439 | HEXB |
| 440 | HFE |
| 441 | HGF |
| 442 | HIF1A |
| 443 | HIP1 |
| 444 | HIPK2 |
| 445 | HIVEP3 |
| 446 | HLA-B |
| 447 | HLA-C |
| 448 | HLA-DMA |
| 449 | HLA-DMB |
| 450 | HLA-DOA |
| 451 | HLA-DPB1 |
| 452 | HLA-DQA1 |
| 453 | HLA-DQA2 |
| 454 | HLA-DQB1 |
| 455 | HLA-DQB2 |
| 456 | HLA-DRA |
| 457 | HLA-DRB1 |
| 458 | HLA-DRB4 |
| 459 | HLA-E |
| 460 | HLA-G |
| 461 | HMGB1 |
| 462 | HMOX1 |
| 463 | HNRNPA2B1 |
| 464 | HOTAIR |
| 465 | HOXD9 |
| 466 | HP |
| 467 | HPSE |
| 468 | HRH4 |
| 469 | HSD11B2 |
| 470 | HSP90AB1 |
| 471 | HSP90B1 |
| 472 | HSPA1A |
| 473 | HSPA5 |
| 474 | HSPA8 |
| 475 | HSPB8 |
| 476 | HSPD1 |
| 477 | HTR2A |
| 478 | IAPP |
| 479 | ICAM1 |
| 480 | ICOS |
| 481 | ID2 |
| 482 | IDDM8 |
| 483 | IER3 |
| 484 | IFI16 |
| 485 | IFN1@ |
| 486 | IFNA2 |
| 487 | IFNG |
| 488 | IFNGR1 |
| 489 | IFNGR2 |
| 490 | IFNK |
| 491 | IFNL1 |
| 492 | IGF1 |
| 493 | IGF1R |
| 494 | IGF2 |
| 495 | IGFBP1 |
| 496 | IGFBP3 |
| 497 | IGHV1-69 |
| 498 | IHH |
| 499 | IKBKE |
| 500 | IKZF2 |
| 501 | IKZF3 |
| 502 | IL10 |
| 503 | IL10RA |
| 504 | IL11 |
| 505 | IL12A |
| 506 | IL12B |
| 507 | IL13 |
| 508 | IL15 |
| 509 | IL15RA |
| 510 | IL16 |
| 511 | IL17A |
| 512 | IL17F |
| 513 | IL17RA |
| 514 | IL17RC |
| 515 | IL18 |
| 516 | IL18R1 |
| 517 | IL1A |
| 518 | IL1B |
| 519 | IL1R1 |
| 520 | IL1RL1 |
| 521 | IL1RN |
| 522 | IL2 |
| 523 | IL20 |
| 524 | IL20RB |
| 525 | IL21 |
| 526 | IL21R |
| 527 | IL22 |
| 528 | IL23A |
| 529 | IL23R |
| 530 | IL24 |
| 531 | IL26 |
| 532 | IL27 |
| 533 | IL2RA |
| 534 | IL2RB |
| 535 | IL3 |
| 536 | IL31 |
| 537 | IL32 |
| 538 | IL33 |
| 539 | IL34 |
| 540 | IL36A |
| 541 | IL36B |
| 542 | IL37 |
| 543 | IL4 |
| 544 | IL4R |
| 545 | IL6 |
| 546 | IL6R |
| 547 | IL6ST |
| 548 | IL7 |
| 549 | IL7R |
| 550 | IL9 |
| 551 | IL9R |
| 552 | INHA |
| 553 | INHBA |
| 554 | INPP5B |
| 555 | IRAK1 |
| 556 | IRAK2 |
| 557 | IRF1 |
| 558 | IRF4 |
| 559 | IRF5 |
| 560 | IRF8 |
| 561 | IRS1 |
| 562 | IRX1 |
| 563 | ITCH |
| 564 | ITGA1 |
| 565 | ITGA4 |
| 566 | ITGA9 |
| 567 | ITGAM |
| 568 | ITGAV |
| 569 | ITGB1 |
| 570 | ITGB2 |
| 571 | ITGB5 |
| 572 | ITLN1 |
| 573 | ITPR2 |
| 574 | JAG1 |
| 575 | JAK2 |
| 576 | JAK3 |
| 577 | JAM3 |
| 578 | JAZF1 |
| 579 | JUN |
| 580 | KCNIP4 |
| 581 | KCNK5 |
| 582 | KCNMA1 |
| 583 | KHDRBS1 |
| 584 | KIAA1109 |
| 585 | KIF5A |
| 586 | KIR2DL1 |
| 587 | KIR2DL2 |
| 588 | KIR2DL3 |
| 589 | KIR2DS2 |
| 590 | KIR2DS4 |
| 591 | KLF12 |
| 592 | KLRB1 |
| 593 | KLRC1 |
| 594 | KLRC2 |
| 595 | KLRD1 |
| 596 | KLRG1 |
| 597 | KLRK1 |
| 598 | KPNA1 |
| 599 | KPNA2 |
| 600 | KREMEN1 |
| 601 | KRT8 |
| 602 | LAIR1 |
| 603 | LAMP3 |
| 604 | LBH |
| 605 | LCE3B |
| 606 | LCE3C |
| 607 | LDLR |
| 608 | LECT2 |
| 609 | LEP |
| 610 | LEPQTL1 |
| 611 | LEPR |
| 612 | LGALS2 |
| 613 | LGALS3 |
| 614 | LGALS9 |
| 615 | LILRA3 |
| 616 | LILRB1 |
| 617 | LINC00452 |
| 618 | LINC01104 |
| 619 | LINC01483 |
| 620 | LINC02656 |
| 621 | LMO4 |
| 622 | LOC100506023 |
| 623 | LOC100506403 |
| 624 | LOC101927437 |
| 625 | LOC730100 |
| 626 | LOXL2 |
| 627 | LPAR1 |
| 628 | LPAR3 |
| 629 | LPIN2 |
| 630 | LRP1 |
| 631 | LRP2 |
| 632 | LRP5 |
| 633 | LRRK2 |
| 634 | LSP1 |
| 635 | LST1 |
| 636 | LTA |
| 637 | LTB |
| 638 | LTB4R |
| 639 | LTB4R2 |
| 640 | LTBR |
| 641 | LVRN |
| 642 | MACIR |
| 643 | MAF |
| 644 | MAFB |
| 645 | MALT1 |
| 646 | MAP2K4 |
| 647 | MAP2K6 |
| 648 | MAP2K7 |
| 649 | MAP3K2 |
| 650 | MAP3K5 |
| 651 | MAP3K8 |
| 652 | MAPK1 |
| 653 | MAPK14 |
| 654 | MAPK3 |
| 655 | MAPK8 |
| 656 | MAPK9 |
| 657 | MBD4 |
| 658 | MBL2 |
| 659 | MBP |
| 660 | MC2R |
| 661 | MC3R |
| 662 | MC4R |
| 663 | MCL1 |
| 664 | MDGA2 |
| 665 | MDK |
| 666 | MDM2 |
| 667 | MECP2 |
| 668 | MED1 |
| 669 | MEFV |
| 670 | MERTK |
| 671 | MFAP4 |
| 672 | MFGE8 |
| 673 | MIA |
| 674 | MIA3 |
| 675 | MICA |
| 676 | MICB |
| 677 | MIF |
| 678 | MIR124-1 |
| 679 | MIR125B1 |
| 680 | MIR126 |
| 681 | MIR132 |
| 682 | MIR140 |
| 683 | MIR143 |
| 684 | MIR145 |
| 685 | MIR146A |
| 686 | MIR155 |
| 687 | MIR155HG |
| 688 | MIR16-1 |
| 689 | MIR17 |
| 690 | MIR188 |
| 691 | MIR1908 |
| 692 | MIR192 |
| 693 | MIR199A1 |
| 694 | MIR21 |
| 695 | MIR210 |
| 696 | MIR212 |
| 697 | MIR218-1 |
| 698 | MIR22 |
| 699 | MIR221 |
| 700 | MIR222 |
| 701 | MIR223 |
| 702 | MIR23A |
| 703 | MIR23B |
| 704 | MIR26B |
| 705 | MIR27A |
| 706 | MIR27B |
| 707 | MIR29A |
| 708 | MIR301A |
| 709 | MIR323A |
| 710 | MIR338 |
| 711 | MIR346 |
| 712 | MIR34A |
| 713 | MIR361 |
| 714 | MIR3614 |
| 715 | MIR363 |
| 716 | MIR371B |
| 717 | MIR3926-1 |
| 718 | MIR425 |
| 719 | MIR451A |
| 720 | MIR4728 |
| 721 | MIR498 |
| 722 | MIR499A |
| 723 | MIR5100 |
| 724 | MIR5196 |
| 725 | MIR532 |
| 726 | MIR573 |
| 727 | MIR613 |
| 728 | MIR650 |
| 729 | MIR671 |
| 730 | MIR92A1 |
| 731 | MITF |
| 732 | MMEL1 |
| 733 | MMP1 |
| 734 | MMP13 |
| 735 | MMP14 |
| 736 | MMP2 |
| 737 | MMP3 |
| 738 | MMP9 |
| 739 | MOB3B |
| 740 | MPG |
| 741 | MPO |
| 742 | MRC1 |
| 743 | MRPL17 |
| 744 | MS4A1 |
| 745 | MSH6 |
| 746 | MSR1 |
| 747 | MSRA |
| 748 | MTDH |
| 749 | MTF1 |
| 750 | MTHFR |
| 751 | MTNR1B |
| 752 | MTOR |
| 753 | MTRR |
| 754 | MUC1 |
| 755 | MUC5B |
| 756 | MUC7 |
| 757 | MUTYH |
| 758 | MYC |
| 759 | MYO9B |
| 760 | N4BP1 |
| 761 | NAA25 |
| 762 | NADSYN1 |
| 763 | NAMPT |
| 764 | NAT2 |
| 765 | NCAM1 |
| 766 | NCF4 |
| 767 | NCOR1 |
| 768 | NCR1 |
| 769 | NCR2 |
| 770 | NCR3 |
| 771 | NEDD9 |
| 772 | NEWENTRY |
| 773 | NFATC1 |
| 774 | NFATC2 |
| 775 | NFKB1 |
| 776 | NFKBIA |
| 777 | NFKBIE |
| 778 | NFKBIL1 |
| 779 | NGFR |
| 780 | NIN |
| 781 | NKAPL |
| 782 | NLRP1 |
| 783 | NLRP2 |
| 784 | NLRP3 |
| 785 | NLRP6 |
| 786 | NMT1 |
| 787 | NOD1 |
| 788 | NOD2 |
| 789 | NONO |
| 790 | NOS2 |
| 791 | NOS3 |
| 792 | NOX1 |
| 793 | NPHS1 |
| 794 | NPPB |
| 795 | NPPC |
| 796 | NPSR1 |
| 797 | NPY |
| 798 | NR1H2 |
| 799 | NR1H3 |
| 800 | NR2F2 |
| 801 | NR3C1 |
| 802 | NR4A2 |
| 803 | NRP1 |
| 804 | NRP2 |
| 805 | NTN1 |
| 806 | NTT |
| 807 | OGG1 |
| 808 | OLAH |
| 809 | OLIG3 |
| 810 | OLR1 |
| 811 | OPRM1 |
| 812 | ORM1 |
| 813 | ORM2 |
| 814 | OS9 |
| 815 | OSCAR |
| 816 | OSM |
| 817 | OSMR |
| 818 | P2RX7 |
| 819 | PADI2 |
| 820 | PADI3 |
| 821 | PADI4 |
| 822 | PARP1 |
| 823 | PCSK6 |
| 824 | PCSK9 |
| 825 | PDCD1 |
| 826 | PDCD1LG2 |
| 827 | PDCD5 |
| 828 | PDE2A |
| 829 | PDE3A |
| 830 | PDIA3 |
| 831 | PDPK1 |
| 832 | PDPN |
| 833 | PECAM1 |
| 834 | PERP |
| 835 | PF4 |
| 836 | PFKFB3 |
| 837 | PFKL |
| 838 | PGK1 |
| 839 | PGLYRP1 |
| 840 | PHACTR3 |
| 841 | PHF19 |
| 842 | PHTF1 |
| 843 | PIAS3 |
| 844 | PIK3CA |
| 845 | PIK3CD |
| 846 | PIK3CG |
| 847 | PIM2 |
| 848 | PIN1 |
| 849 | PIP4K2C |
| 850 | PIWIL2 |
| 851 | PIWIL4 |
| 852 | PLA2G2A |
| 853 | PLA2G4A |
| 854 | PLA2G7 |
| 855 | PLAU |
| 856 | PLAUR |
| 857 | PLB1 |
| 858 | PLCL2 |
| 859 | PLD4 |
| 860 | PLEK |
| 861 | PLTP |
| 862 | PML |
| 863 | PON1 |
| 864 | PON2 |
| 865 | POU2AF1 |
| 866 | POU3F1 |
| 867 | PPARG |
| 868 | PPARGC1B |
| 869 | PPBP |
| 870 | PPIL4 |
| 871 | PPM1L |
| 872 | PRDM1 |
| 873 | PRG4 |
| 874 | PRKAA1 |
| 875 | PRKCB |
| 876 | PRKCD |
| 877 | PRKCH |
| 878 | PRKCQ |
| 879 | PRKCZ |
| 880 | PRKDC |
| 881 | PRL |
| 882 | PRLR |
| 883 | PRMT5 |
| 884 | PROC |
| 885 | PRRC2A |
| 886 | PRSS2 |
| 887 | PRTN3 |
| 888 | PSMA4 |
| 889 | PSMB7 |
| 890 | PSMB9 |
| 891 | PSME3 |
| 892 | PSTPIP1 |
| 893 | PTGER2 |
| 894 | PTGER4 |
| 895 | PTGES |
| 896 | PTGS1 |
| 897 | PTGS2 |
| 898 | PTH |
| 899 | PTH1R |
| 900 | PTK2 |
| 901 | PTPN11 |
| 902 | PTPN2 |
| 903 | PTPN22 |
| 904 | PTPRC |
| 905 | PTPRJ |
| 906 | PTPRK |
| 907 | PTPRM |
| 908 | PTPRS |
| 909 | PTX3 |
| 910 | PVT1 |
| 911 | PXK |
| 912 | QKI |
| 913 | QPCT |
| 914 | RABEP1 |
| 915 | RAC1 |
| 916 | RAD51B |
| 917 | RAF1 |
| 918 | RAG1 |
| 919 | RAG2 |
| 920 | RAMP2 |
| 921 | RAP1A |
| 922 | RARRES2 |
| 923 | RASGRP1 |
| 924 | RASGRP2 |
| 925 | RASGRP4 |
| 926 | RB1 |
| 927 | RBP4 |
| 928 | RBPJ |
| 929 | RCHY1 |
| 930 | REG1A |
| 931 | REL |
| 932 | RELA |
| 933 | RELN |
| 934 | RETN |
| 935 | REV3L |
| 936 | RHOA |
| 937 | RIT2 |
| 938 | RNASE2 |
| 939 | RNASEH2B |
| 940 | ROBO1 |
| 941 | ROBO3 |
| 942 | RORC |
| 943 | RPP14 |
| 944 | RPS12P4 |
| 945 | RPS6KA3 |
| 946 | RPS6KA4 |
| 947 | RPS6KA5 |
| 948 | RTKN2 |
| 949 | RUNX1 |
| 950 | S100A11 |
| 951 | S100A12 |
| 952 | S100A4 |
| 953 | S100A8 |
| 954 | S100A9 |
| 955 | S1PR1 |
| 956 | S1PR2 |
| 957 | S1PR3 |
| 958 | SAA1 |
| 959 | SALL3 |
| 960 | SAP30BP |
| 961 | SCARB1 |
| 962 | SDC3 |
| 963 | SELE |
| 964 | SELENOS |
| 965 | SELP |
| 966 | SEMA3A |
| 967 | SEMA3C |
| 968 | SEMA4A |
| 969 | SEMA4D |
| 970 | SEMA7A |
| 971 | SEMA7A |
| 972 | SERPINE1 |
| 973 | SERPINF1 |
| 974 | SERPINH1 |
| 975 | SFRP5 |
| 976 | SFTPA1 |
| 977 | SFTPB |
| 978 | SFTPC |
| 979 | SFTPD |
| 980 | SH2B3 |
| 981 | SH2D2A |
| 982 | SH3PXD2A |
| 983 | SH3RF1 |
| 984 | SHH |
| 985 | SIAE |
| 986 | SIGIRR |
| 987 | SIGLEC1 |
| 988 | SIGLEC9 |
| 989 | SIRT1 |
| 990 | SIRT6 |
| 991 | SLAMF7 |
| 992 | SLC11A1 |
| 993 | SLC16A4 |
| 994 | SLC19A1 |
| 995 | SLC22A4 |
| 996 | SLC22A5 |
| 997 | SLC2A3 |
| 998 | SLC30A6 |
| 999 | SLC6A11 |
| 1000 | SLC7A5 |
| 1001 | SLC8A3 |
| 1002 | SLCO1C1 |
| 1003 | SLIT3 |
| 1004 | SLPI |
| 1005 | SMAD3 |
| 1006 | SMAD7 |
| 1007 | SMIM21 |
| 1008 | SMO |
| 1009 | SMTNL2 |
| 1010 | SNAI2 |
| 1011 | SNAPIN |
| 1012 | SOCS1 |
| 1013 | SOD1 |
| 1014 | SOST |
| 1015 | SOX4 |
| 1016 | SOX5 |
| 1017 | SPA17 |
| 1018 | SPAG16 |
| 1019 | SPARC |
| 1020 | SPHK1 |
| 1021 | SPHK2 |
| 1022 | SPI1 |
| 1023 | SPP1 |
| 1024 | SPRED2 |
| 1025 | SPRY2 |
| 1026 | SPSB1 |
| 1027 | SREBF1 |
| 1028 | ST2 |
| 1029 | STAG1 |
| 1030 | STAT3 |
| 1031 | STAT4 |
| 1032 | STAT5A |
| 1033 | STEAP4 |
| 1034 | STXBP6 |
| 1035 | SUCNR1 |
| 1036 | SUMO1 |
| 1037 | SUMO4 |
| 1038 | SUOX |
| 1039 | SYK |
| 1040 | SYNGR1 |
| 1041 | SYVN1 |
| 1042 | TAB2 |
| 1043 | TAC1 |
| 1044 | TACR1 |
| 1045 | TAGAP |
| 1046 | TAP1 |
| 1047 | TAP2 |
| 1048 | TBX21 |
| 1049 | TBX5 |
| 1050 | TCTA |
| 1051 | TEC |
| 1052 | TEK |
| 1053 | TERT |
| 1054 | TESPA1 |
| 1055 | TF |
| 1056 | TGFB1 |
| 1057 | TGFBI |
| 1058 | THBS1 |
| 1059 | THOP1 |
| 1060 | TIA1 |
| 1061 | TIMD4 |
| 1062 | TIMP1 |
| 1063 | TIMP3 |
| 1064 | TIRAP |
| 1065 | TLR10 |
| 1066 | TLR2 |
| 1067 | TLR3 |
| 1068 | TLR4 |
| 1069 | TLR5 |
| 1070 | TLR7 |
| 1071 | TLR8 |
| 1072 | TLR9 |
| 1073 | TMEM187 |
| 1074 | TNC |
| 1075 | TNF |
| 1076 | TNFAIP3 |
| 1077 | TNFAIP6 |
| 1078 | TNFRSF10A |
| 1079 | TNFRSF10B |
| 1080 | TNFRSF10D |
| 1081 | TNFRSF11A |
| 1082 | TNFRSF11B |
| 1083 | TNFRSF12A |
| 1084 | TNFRSF13C |
| 1085 | TNFRSF14 |
| 1086 | TNFRSF17 |
| 1087 | TNFRSF18 |
| 1088 | TNFRSF1A |
| 1089 | TNFRSF1B |
| 1090 | TNFRSF25 |
| 1091 | TNFRSF4 |
| 1092 | TNFRSF6B |
| 1093 | TNFRSF8 |
| 1094 | TNFRSF9 |
| 1095 | TNFSF10 |
| 1096 | TNFSF11 |
| 1097 | TNFSF12 |
| 1098 | TNFSF13 |
| 1099 | TNFSF13B |
| 1100 | TNFSF14 |
| 1101 | TNFSF15 |
| 1102 | TNFSF18 |
| 1103 | TNFSF4 |
| 1104 | TNFSF8 |
| 1105 | TNFSF9 |
| 1106 | TNIP1 |
| 1107 | TNIP3 |
| 1108 | TNNI3 |
| 1109 | TNNT2 |
| 1110 | TNPO3 |
| 1111 | TP53 |
| 1112 | TP53AIP1 |
| 1113 | TPD52 |
| 1114 | TPMT |
| 1115 | TPSAB1 |
| 1116 | TPT1 |
| 1117 | TRAF1 |
| 1118 | TRAF2 |
| 1119 | TRAF3IP2 |
| 1120 | TRAF6 |
| 1121 | TRAIP |
| 1122 | TRB |
| 1123 | TREM1 |
| 1124 | TREM2 |
| 1125 | TREX1 |
| 1126 | TRHDE |
| 1127 | TRIB1 |
| 1128 | TRIB2 |
| 1129 | TRIB3 |
| 1130 | TRIM3 |
| 1131 | TRIM38 |
| 1132 | TRPC1 |
| 1133 | TRPC5 |
| 1134 | TRPV1 |
| 1135 | TSBP1 |
| 1136 | TSLP |
| 1137 | TTC38 |
| 1138 | TXNDC11 |
| 1139 | TXNDC5 |
| 1140 | TXNRD1 |
| 1141 | TYK2 |
| 1142 | TYMS |
| 1143 | TYRO3 |
| 1144 | UBASH3A |
| 1145 | UBE2L3 |
| 1146 | UCA1 |
| 1147 | UNC13D |
| 1148 | UNC5B |
